# Slow Ca^2+^ Efflux by Ca^2+^/H^+^ Exchange in Cardiac Mitochondria Is Modulated by Ca^2+^ Re-uptake via MCU, Extra-Mitochondrial pH, and H^+^ Pumping by F_O_F_1_-ATPase

**DOI:** 10.3389/fphys.2018.01914

**Published:** 2019-02-04

**Authors:** Johan Haumann, Amadou K. S. Camara, Ashish K. Gadicherla, Christopher D. Navarro, Age D. Boelens, Christoph A. Blomeyer, Ranjan K. Dash, Michael R. Boswell, Wai-Meng Kwok, David F. Stowe

**Affiliations:** ^1^Department of Anesthesiology, Medical College of Wisconsin, Milwaukee, WI, United States; ^2^Department of Physiology, Medical College of Wisconsin, Milwaukee, WI, United States; ^3^Cardiovascular Center, Medical College of Wisconsin, Milwaukee, WI, United States; ^4^Cancer Center, Medical College of Wisconsin, Milwaukee, WI, United States; ^5^Department of Biomedical Engineering, Medical College of Wisconsin and Marquette University, Milwaukee, WI, United States; ^6^Department of Pharmacology and Toxicology, Medical College of Wisconsin, Milwaukee, WI, United States; ^7^Research Service, Veterans Affairs Medical Center, Milwaukee, WI, United States

**Keywords:** cardiac mitochondria, Ca^2+^ uptake/release, mitochondrial Ca^2+^ uniporter, Ca^2+^/H^+^ exchange, H^+^ leak and pumping, complex V

## Abstract

Mitochondrial (m) Ca^2+^ influx is largely dependent on membrane potential (ΔΨ_m_), whereas mCa^2+^ efflux occurs primarily via Ca^2+^ ion exchangers. We probed the kinetics of Ca^2+^/H^+^ exchange (CHE_m_) in guinea pig cardiac muscle mitochondria. We tested if net mCa^2+^ flux is altered during a matrix inward H^+^ leak that is dependent on matrix H^+^ pumping by ATP_m_ hydrolysis at complex V (F_O_F_1_-ATPase). We measured [Ca^2+^]_m_, extra-mitochondrial (e) [Ca^2+^]_e_, ΔΨ_m_, pH_m_, pH_e_, NADH, respiration, ADP/ATP ratios, and total [ATP]_m_ in the presence or absence of protonophore dinitrophenol (DNP), mitochondrial uniporter (MCU) blocker Ru360, and complex V blocker oligomycin (OMN). We proposed that net slow influx/efflux of Ca^2+^ after adding DNP and CaCl_2_ is dependent on whether the ΔpH_m_ gradient is/is not maintained by reciprocal outward H^+^ pumping by complex V. We found that adding CaCl_2_ enhanced DNP-induced increases in respiration and decreases in ΔΨ_m_ while [ATP]_m_ decreased, ΔpH_m_ gradient was maintained, and [Ca^2+^]_m_ continued to increase slowly, indicating net mCa^2+^ influx via MCU. In contrast, with complex V blocked by OMN, adding DNP and CaCl_2_ caused larger declines in ΔΨ_m_ as well as a slow fall in pH_m_ to near pH_e_ while [Ca^2+^]_m_ continued to decrease slowly, indicating net mCa^2+^ efflux in exchange for H^+^ influx (CHE_m_) until the ΔpH_m_ gradient was abolished. The kinetics of slow mCa^2+^ efflux with slow H^+^ influx via CHE_m_ was also observed at pH_e_ 6.9 vs. 7.6 by the slow fall in pH_m_ until ΔpH_m_ was abolished; if Ca^2+^ reuptake via the MCU was also blocked, mCa^2+^ efflux via CHE_m_ became more evident. Of the two components of the proton electrochemical gradient, our results indicate that CHE_m_ activity is driven largely by the ΔpH_m_ chemical gradient with H^+^ leak, while mCa^2+^ entry via MCU depends largely on the charge gradient ΔΨ_m_. A fall in ΔΨ_m_ with excess mCa^2+^ loading can occur during cardiac cell stress. Cardiac cell injury due to mCa^2+^ overload may be reduced by temporarily inhibiting F_O_F_1_-ATPase from pumping H^+^ due to ΔΨ_m_ depolarization. This action would prevent additional slow mCa^2+^ loading via MCU and permit activation of CHE_m_ to mediate efflux of mCa^2+^.

**HIGHLIGHTS**

- We examined how slow mitochondrial (m) Ca^2+^ efflux via Ca^2+^/H^+^ exchange (CHE_m_) is triggered by matrix acidity after a rapid increase in [Ca^2+^]_m_ by adding CaCl_2_ in the presence of dinitrophenol (DNP) to permit H^+^ influx, and oligomycin (OMN) to block H^+^ pumping via F_O_F_1_-ATP synthase/ase (complex V).
- Declines in ΔΨ_m_ and pH_m_ after DNP and added CaCl_2_ were larger when complex V was blocked.
- [Ca^2+^]_m_ slowly increased despite a fall in ΔΨ_m_ but maintained pH_m_ when H^+^ pumping by complex V was permitted.
- [Ca^2+^]_m_ slowly decreased and external [Ca^2+^]_e_ increased with declines in both ΔΨ_m_ and pH_m_ when complex V was blocked.
- ATP_m_ hydrolysis supports a falling pH_m_ and redox state and promotes a slow increase in [Ca^2+^]_m_.
- After rapid Ca^2+^ influx due to a bolus of CaCl_2_, slow mCa^2+^ efflux by CHE_m_ occurs directly if pH_e_ is low.

## Introduction

Mitochondrial (m) Ca^2+^ overload is a damaging consequence of cardiac ischemia-reperfusion (IR) injury and hence is an important subject for potential therapy (Brookes et al., 2004; O’Rourke et al., 2005; Stowe and Camara, 2009; Camara et al., 2010). During IR, mitochondria can consume rather than generate ATP (Chinopoulos and Adam-Vizi, 2010; Chinopoulos et al., 2010), which consequently can augment mCa^2+^ overload (Riess et al., 2002) sufficient to induce cell apoptosis and necrosis (Murphy and Steenbergen, 2008). [Ca^2+^]_m_ is regulated in part by electrochemical dependent cation flux via Ca^2+^ transporters and by cation exchangers within the inner mitochondrial membrane (IMM) (Gunter and Pfeiffer, 1990; Gunter et al., 1994; Bernardi, 1999; Brookes et al., 2004). The major route for mCa^2+^ uptake is via the ruthenium red (RR) sensitive mitochondrial Ca^2+^ uniporter (MCU), now considered a macromolecular complex composed of two pore components, MCU and MCUb, and MCU regulators MCU1, 2, 3, and EMRE (essential MCU regulator), and other components (De Stefani et al., 2015). Ca^2+^ influx via the MCU is reduced by competition with cytosolic Mg^2+^ (Boelens et al., 2013; Tewari et al., 2014). Additional modes of mCa^2+^ uptake are proposed to occur via a ryanodine type channel (RTC) in the IMM (Ryu et al., 2011; O-Uchi et al., 2013; Tewari et al., 2014) and at the sarcoplasmic reticular-MCU interface where functional Ca^2+^ signaling between the cytoplasmic and mitochondrial compartments is believed to occur (Csordas et al., 2010).

A primary mCa^2+^ efflux pathway is the Na^+^/Ca^2+^ exchanger (NCE_m_) (Boyman et al., 2013). In unicellular organisms and in some non-cardiac tissues there is firm evidence (Azzone et al., 1977; Pozzan et al., 1977; Wingrove et al., 1984; Brand, 1985; Rottenberg and Marbach, 1990; Gunter et al., 1991, 1994; Bernardi, 1999; Demaurex et al., 2009; Nishizawa et al., 2013) for slow homeostatic mCa^2+^ efflux through a Na^+^-independent Ca^2+^ exchanger (NICE), i.e., a non-electrogenic Ca^2+^/H^+^ exchanger (CHE) that might be activated when the ΔpH_m_ gradient across the IMM is altered. The amount of free (ionized) [Ca^2+^]_m_ available for exchange depends on the extent of dynamic mCa^2+^ buffering (Bazil et al., 2013; Blomeyer et al., 2013; Tewari et al., 2014). mCa^2+^ influx via the MCU and efflux via the NCE_m_ are largely voltage (ΔΨ_m_) dependent, whereas Ca^2+^ transport via the CHE_m_, while pH-dependent, may be electrogenic (1 H^+^ for 1 Ca^2+^) or non-electrogenic (2 H^+^ for 1 Ca^2+^). However, CHE_m_ can be indirectly dependent on the full IMM electrochemical gradient if there is a decrease in the IMM ΔpH_m_ gradient (Rottenberg and Marbach, 1990; Dash and Beard, 2008; Dash et al., 2009).

There is a well-known direct correlation between ΔΨ_m_ and mCa^2+^ uptake based on the Nernst equation; a more polarized ΔΨ_m_ permits greater mCa^2+^ uptake (Wingrove et al., 1984; Gunter et al., 1994). mCa^2+^ uptake via the MCU depends both on the electrical (charge) gradient, ΔΨ_m_, and on the concentration gradient for [Ca^2+^] across the IMM. ATP_m_ hydrolysis with H^+^ pumping can occur at complex V (F_O_F_1_-ATPsynthase/ase) during cardiac ischemia (Jennings et al., 1991) in an attempt to maintain the ΔpH_m_ gradient, and along with the ΔΨ_m_ gradient (Chinopoulos and Adam-Vizi, 2010; Chinopoulos, 2011), equals the proton motive force, *pmf*. However, it is not known how the magnitude, rate, and route of mCa^2+^ uptake or release in cardiac muscle cell mitochondria is affected by manipulating the IMM Δ[H^+^]_m_ gradient by allowing mATP hydrolysis, which would result in H^+^ pumping and better maintain the Δ[H^+^] gradient when ΔΨ_m_ is low, vs. blocking mATP hydrolysis (no H^+^ pumping with collapsing Δ[H^+^]) and lower ΔΨ_m_.

Exposure of mitochondria to external (e) CaCl_2_ when the IMM is fully charged (high ΔΨ_m_), defined here by the presence of substrate in state 2 conditions without an induced inward H^+^ leak, promotes rapid voltage-dependent mCa^2+^ uptake via MCU (Hoppe, 2010). In contrast, decreased net mCa^2+^ uptake might be expected during a protonophore-induced inward H^+^ leak if H^+^ influx leads to Ca^2+^ efflux. However, an inward H^+^ flux that slowly decreases ΔΨ_m_ can still result in a slow, continued uptake of mCa^2+^ via the MCU if there remains sufficient ΔΨ_m_ and Ca^2+^ chemical gradient ([Ca^2+^]_e_ > [Ca^2+^]_m_) across the IMM. mCa^2+^ influx via the MCU can partially depolarize ΔΨ_m_ (Delcamp et al., 1998; Di Lisa and Bernardi, 1998) due to the influx of positive charges without an effect on the Δ[H^+^]_m_, and more so with a fall in Δ[H^+^]_m_ gradient from the added influx of H^+^ in the presence of a protonophore.

Our aim was to mechanistically examine the slow mode kinetics of mCa^2+^ influx/efflux in cardiac cell mitochondria. The conditions under which CHE_m_ may occur in cardiac mitochondria are unknown. We proposed that an induced, net influx of H^+^ is coupled to net mCa^2+^ efflux by activation of CHE_m_ in the face of continued mCa^2+^ uptake via the MCU in partially depolarized ΔΨ_m_ mitochondria. In addition, if the extra-mitochondrial milieu is acidic, pH_m_ would slowly decrease as mH^+^ entry by mCHE_m_ is exchanged for mCa^2+^ efflux in Ca^2+^ overloaded mitochondria. We postulated that CHE_m_ is activated under conditions of slow a H^+^ influx and a high m[Ca^2+^], and especially when H^+^ pumping by complex V, stimulated by the lowered ΔΨ_m,_ is prevented. To carry out our aim, we examined the time dependent changes in ΔΨ_m_, [Ca^2+^]_m_ and pH_m_, and extra-mitochondrial [Ca^2+^]_e_ and pH_e_, after a bolus of CaCl_2_ either by inducing an inward H^+^ leak that causes an outward pumping of H^+^ by complex V, or by altering the extra-mitochondrial pH_e_.

In one set of experiments, we challenged isolated energized mitochondria with a bolus of CaCl_2_ in the absence or presence of increasing concentrations of the protonophore 2,4-dinitrophenol (DNP) in the absence or presence of the complex V inhibitor oligomycin (OMN) to block ATP hydrolysis-induced H^+^ pumping, and or Ru360 to block the reuptake of Ca^2+^ via the MCU. To understand how DNP, OMN, and Ru360 dynamically alter [Ca^2+^]_m_ or [Ca^2+^]_e_ after a bolus of CaCl_2_, we considered it crucial to also dynamically measure ΔΨ_m_, pH_m_, and NADH, as well as mitochondrial respiration (extent of uncoupling), total [ATP]_m_, and ATP_m_/ADP_m_ ratio. In another set of isolated mitochondrial experiments, we directly induced mCa^2+^ efflux via CHE_m_ after CaCl_2_ loading by altering the Na^+^-free medium from a control pH_e_ of 7.15 to either pH 7.6 or 6.9. We show that secondary Ca^2+^ influx vs. efflux is Δ[H^+^]_m_ dependent.

## Materials and Methods

### Isolated Mitochondrial Experiments

All experiments conformed to the Guide for the Care and Use of Laboratory Animal and were approved by the Medical College of Wisconsin Biomedical Resource Center animal studies committee. Detailed methods for mitochondrial isolation and measurements of ΔΨ_m_, [Ca^2+^]_m_, NADH redox state, pH_m_, [ATP]_m_, ADP_m_/ATP_m_ ratio, respiration, and the number of animals per group, are furnished (see section “Supplementary Materials S.1.1–S.1.12”). Briefly, mitochondria were isolated from guinea pig heart ventricles in iced buffer and were suspended in experimental buffer containing in mM: KCl 130, K_2_HPO_4_ 5, MOPS 20, bovine serum albumin 0.016 and EGTA ∼0.036–0.040 at pH 7.15 (adjusted with KOH) at room temperature (21°C). The experimental buffer had a final protein concentration of 0.5 mg/mL. Specific fluorescent probes and spectrophotometry (Qm-8, Photon Technology International, Birmingham, NJ, United States) were used to measure [Ca^2+^]_m_ (indo-1AM) and buffer [Ca^2+^]_e_ (indo-1 or Fura 4 F penta-K^+^ salt), NADH, an indicator of mitochondrial redox state (autofluorescence), pH_m_ (BCECF-AM), and mitochondrial membrane potential (ΔΨ_m_) assessed by rodamine-123 or TMRM (Heinen et al., 2007; Huang et al., 2007; Aldakkak et al., 2010; Haumann et al., 2010) (all fluorescence probes from Invitrogen^TM^ – Thermo Fisher Scientific). Respiration (Clark electrode) and ATP_m_ (bioluminescence) and ATP_m_/ADP_m_ ratio (HPLC, luminometry) were also measured. The experimental buffer, mitochondrial substrates, and drugs were Na^+^-free to prevent activation of NCE_m_ by extra-mitochondrial Na^+^. The inactivity of the NCE was verified by comparing data from these experiments to data from experiments with added CGP-37157, a known mitochondrial NCE_m_ inhibitor (data not shown).

### Experimental Protocols

#### Medium pH_e_-Induced Changes in pH_m_

**FIGURE 1:**
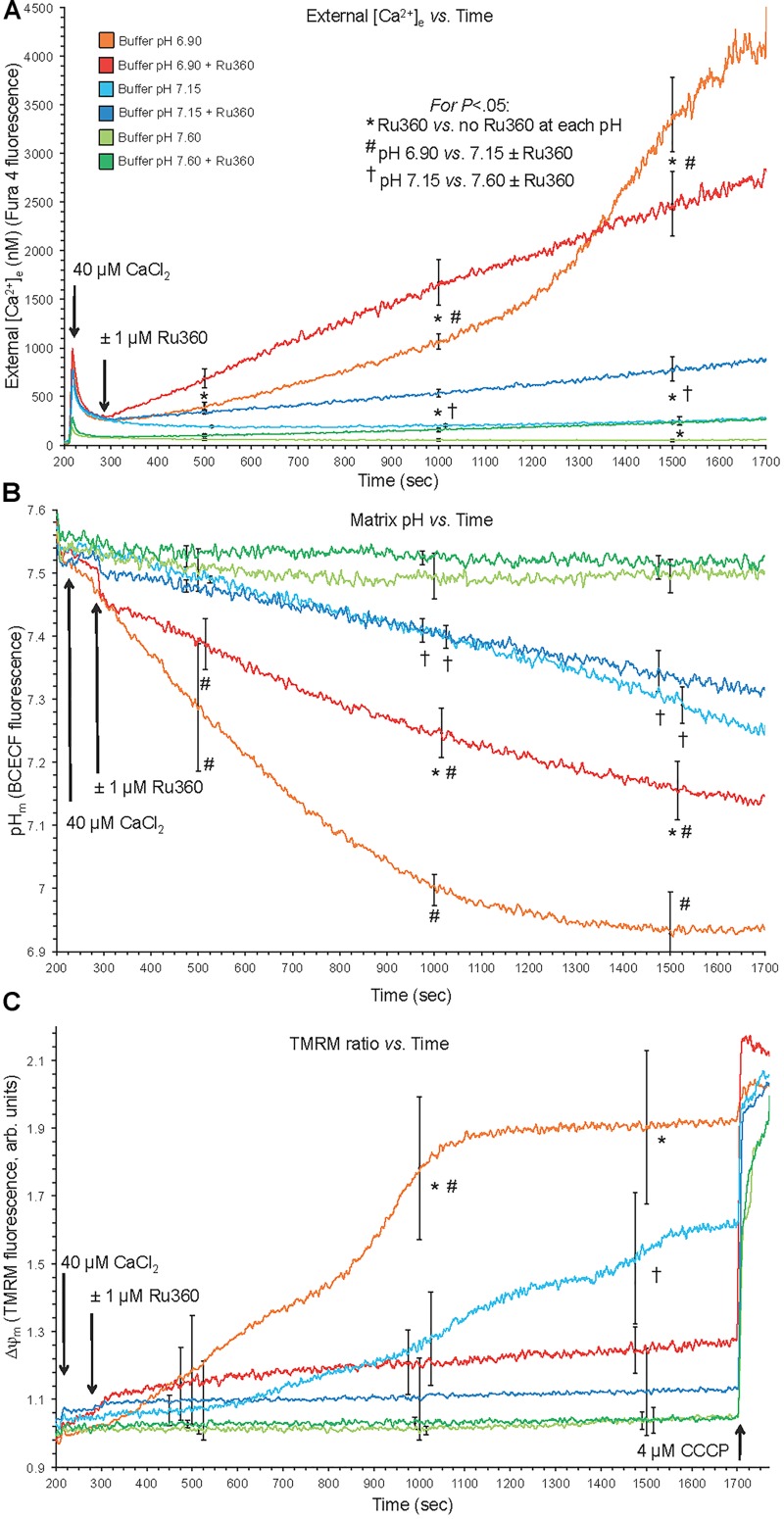
Changes in buffer [Ca^2+^]_e_ **(A)**, matrix pH_m_ **(B)**, and ΔΨ_m_ **(C)** over time after adding 40 μM CaCl_2_ (210 s) at extra-mitochondrial pH_e_ 7.6, 7.15, and 6.9 with or without 1 μM Ru360 (300 s) to inhibit additional mCa^2+^ uptake via MCU. Note the rapid fall in [Ca^2+^]_e_ due to fast mCa^2+^ uptake via the MCU and the following slow rise in [Ca^2+^]_e_ (Ca^2+^ efflux) **(A)**, slow decline in pH_m_ **(B)**, and slow depolarization of ΔΨ_m_ **(C)** at pH 6.9 (each line = mean of 3–4 replicates from 12 guinea pig hearts for each fluorescence measurement). Note in the pH 6.9 medium the faster rate of mCa^2+^ efflux **(A)** over time when MCU was blocked, and the faster declines in pH_m_ **(B)** and ΔΨ_m_ **(C)** over time when MCU was not blocked.

**FIGURE 2:**
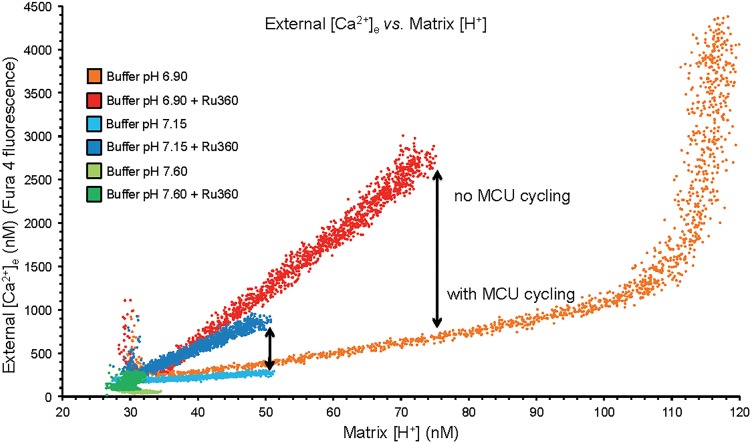
Plots of extra-mitochondrial [Ca^2+^]_e_ as a function of [H^+^]_m_ from the mean data of Figure 1A,B. Arrows denote the difference in net mCa^2+^ efflux at a given [H^+^]_m_ when MCU was or was not blocked with Ru360 given 90 s after the CaCl_2_ bolus. While mCa^2+^ was extruded by CHE_m_, much of it re-entered via the MCU when it was not blocked. At trans-membrane pH equilibrium (pH 6.9) Ca^2+^ uptake via MCU stopped as the ΔΨ_m_ was nearly depolarized having resulted in a large net extrusion of Ca^2+^.

The experimental buffer was identical to that described above except that in addition to the pH 7.15 buffer, buffers at pH 6.9 and 7.6 were prepared by titration with HCl and KOH, respectively. The residual EGTA carried over from the isolation buffer to the experimental buffer resulted in an ionized extra-mitochondrial [Ca^2+^]_e_ of <200 nM (Figure 1). To measure changes in [Ca^2+^]_e_ after adding a bolus of 40 μM CaCl_2_, each pH buffer contained Fura 4 F penta-K^+^ salt. The K_D_’s for Ca^2+^ were calculated and corrected for each buffer pH because pH affects the binding of Ca^2+^ to the fluorescence dye (see section “Supplementary Materials S.1.4, S.1.8”). In other experiments, pH_m_ and ΔΨ_m_ were measured using BCECF-AM and TMRM fluorescent dyes, respectively. Experiments were initiated at *t* = 30 s when mitochondria were added to the buffer; at *t* = 90 s pyruvic acid (PA, 0.5 mM) was added, followed by a bolus of 40 μM CaCl_2_ at *t* = 210 s to initiate rapid mCa^2+^ uptake via MCU. Note that in guinea pig cardiac mitochondria, the respiratory control index (RCI) is higher in the presence of pyruvate alone (Heinen et al., 2007; Blomeyer et al., 2013; Boelens et al., 2013) than with pyruvate plus malate (Riess et al., 2008). For some experiments, 1 μM Ru360 (or vehicle, 0.1% DMSO) was added at *t* = 300 s shortly after adding CaCl_2_ to block Ca^2+^ reuptake into mitochondria via MCU after the Ca^2+^ was extruded from mitochondria. At the end (1700 s) of each experiment, the potent protonophore, carbonyl cyanide m-chlorophenyl hydrazone (CCCP, 4 μM) was given to completely abolish the ΔpH gradient and depolarize ΔΨ_m_. Data for each pH group were collected in mitochondrial suspensions from the same heart; approximately 8–10 hearts were used for each fluorescent probe. At pH 7.15, adding 40 μM CaCl_2_, which increased extra-mitochondrial [Ca^2+^]_e_ into the 1 μM range and increased the initial [Ca^2+^]_m_ to approximately 500 nM (Figure 1, 2), is unlikely to induce membrane permeability transition pore (mPTP) opening. However, to test the possibility of mPTP opening, 500 nM cyclosporine A (CsA), a modulator of cyclophilin D required to open mPTP, was given before adding CaCl_2_ in several experiments at pH_e_ 6.9 and 7.15.

#### Protonophore-Induced Changes in pH_m_

**FIGURE 3:**
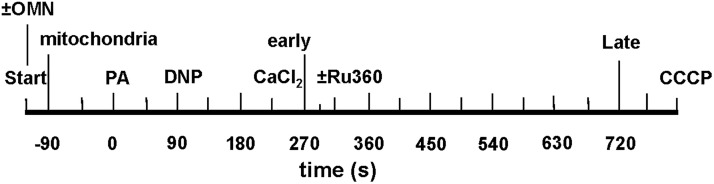
Time line of protonophore-induced experimental protocol: addition of mitochondria, ±oligomycin (OMN), pyruvic acid (PA), dinitrophenol (DNP), CaCl_2_, and CCCP to respiratory buffer. Early and late plots refer to time points where means of several variables are plotted to summarize their interrelationships. In Supplementary Experiments, Ru360 (100 nM) was given to block the MCU (see section “Supplementary Results S.2.4” and Supplementary Figures S.6A,B).

Experiments were initiated at *t* = -120 s; at *t* = -90 s, mitochondria were added to the experimental buffer (time line, Figure 3); external pH_e_ was 7.15. At *t* = 0 s, pyruvic acid (PA, 0.5 mM) was added to the mitochondria suspended in the experimental buffer, followed by 0, 10, 20, 30, or 100 μM DNP, a mild protonophore, at *t* = 90 s, followed by the addition of de-ionized H_2_O, 10, or 25 μM CaCl_2_ at *t* = 225 s. The 90 s period allowed for full ΔΨ_m_ polarization and stabilization of pH_m_ and NADH. In some experiments (see section “Supplementary Results S.2.4” and Supplementary Figure S.6), 100 nM Ru360 was added at *t* = 300 s, after the addition of CaCl_2_, to block any reuptake of mCa^2+^ by the MCU that was extruded by CHE_m_. For the OMN treated groups, 10 μM OMN was added to the experimental buffer at the start of the experimental protocol (Figure 3). At the end of each experiment CCCP was added at *t* = 760 s to maximally depolarize ΔΨ_m_. DNP, Ru360, OMN, and CCCP were each dissolved initially in DMSO and then in buffer to yield a final buffer concentration for DMSO of 0.1 to 0.4% (wt/vol). Each drug or DMSO alone was added to a final volume of 10 μL. To test for mPTP opening, CsA was given before adding 20 or 30 μM DNP and 25 μM CaCl_2_ in several experiments conducted at pH_e_ 7.15.

### Statistical Analyses

**FIGURE 4:**
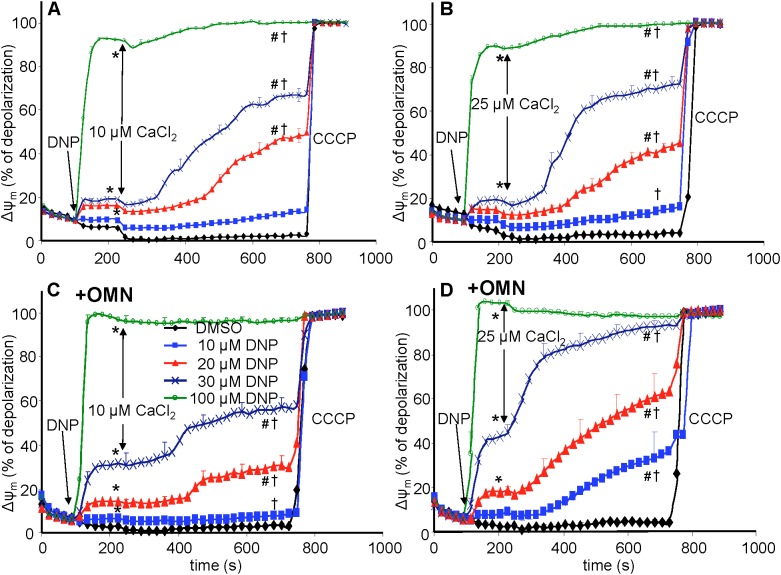
Change in mitochondrial membrane potential (ΔΨ_m_), assessed with rhodamine-123 as % of maximal depolarization, as a function of time after adding dinitrophenol (DNP) and CaCl_2_ in the absence **(A,B)** and presence **(C,D)** of oligomycin (OMN). Note that adding DNP caused a concentration-dependent fall in ΔΨ_m_ that was more pronounced in the presence of OMN. Adding CaCl_2_ caused a small polarization, while increasing depolarization occurred over time. Adding 25 μM CaCl_2_ **(B,D)** resulted in a more pronounced fall in ΔΨ_m_ compared to 10 μM CaCl_2_ **(A,C)**. Buffer pH = 7.15. Data obtained from 10 hearts with 4–5 replicates per heart. For *P* < 0.05: ^∗^after DNP vs. before DNP; ^#^after CaCl_2_ vs. before CaCl_2_; ^†^late (700 s) vs. early (215 s) after CaCl_2_.

Data were summarized at 500, 1000, and 1500 s (for Figure 1, 2) for external buffer-induced changes in pH_m_ on [Ca^2+^]_e_. Data were summarized for protonophore-induced changes in pH_m_ on [Ca^2+^]_m_ at 80 s (after adding PA), 215 s (after adding DNP), 275 s (early after adding CaCl_2_), and 700 s (late after adding CaCl_2_) (e.g., Figure 4). All data points were presented and expressed as average ± SEM. Repeated measure ANOVAs followed by a *post hoc* analyses using Student-Newman-Keuls’ test was performed to determine statistically significant differences among groups. A *P*-value < 0.05 (two-tailed) was considered significant. See Figure legends for statistical notations.

## Results

### CHE_m_ Activation Was Exposed by Efflux of Ca^2+^ With Influx of H^+^ and Was Greater If MCU Was Inhibited

Direct evidence for CHE_m_ activation was observed by acidifying the extra-mitochondrial buffer (low pH_e_), which subsequently decreased the matrix pH_m_ slowly over time (Figure 1). With NCE_m_ and Na^+^/H^+^ (NHE_m_) inactivated by using Na^+^-free solutions and substrates, fast mCa^2+^ influx via the MCU, induced after adding 40 μM CaCl_2_ at pH 6.9, was followed by a slow mCa^2+^ efflux over time ∼(300–1700 s) as shown by the increase in extra-mitochondrial [Ca^2+^]_e_ from <200 nM to nearly 4500 nM in the absence of Ru360 (Figure 1A). When Ru360 was added 90 s after adding CaCl_2_, [Ca^2+^]_e_ rose even more over the first 1000 s, indicating blockade of Ca^2+^ recycling via the MCU and revealing the total mCa^2+^ effluxed via CHE_m_. In the pH 6.9 plus Ru360 group the mean rate (slope) of increase in [Ca^2+^]_e_ (mCa^2+^ efflux) over time (300–1700 s) was 1.5 ± 0.1 nM/s, ΔpH 0.4 units). This was greater than in the pH 6.9 minus Ru360 group (1.0 ± 0.2 nM/s over 300–1000 s), suggesting that approximately 1/3 of the mCa^2+^ extruded was retaken up across the IMM via the MCU. In contrast, mCa^2+^ efflux was not observed in the pH 7.6 medium without Ru360, and minimally at 1500 s at pH 7.6 with Ru360. There was less mCa^2+^ efflux at pH 7.15 ± Ru360 compared to pH 6.9 ± Ru360. However, even at pH 7.15 ± Ru360, there were similar steady declines in pH_e_ while net slow Ca^2+^ efflux was noted only in the plus Ru360 groups, indicating Ca^2+^ re-uptake via MCU. Therefore, in the acidic extra-mitochondrial medium, slow decreases in pH_m_ (H^+^ influx) were accompanied by slow increases in mCa^2+^ efflux, indicating CHE_m_ activity. Eventually, matrix acidification was more pronounced in the pH 6.9 medium (ΔpH 0.62 units) in the absence of Ru360 than in all other groups so that over time as H^+^ influx was exchanged for Ca^2+^ efflux the IMM ΔpH gradient was eventually obliterated, halting Ca^2+^ efflux (Figure 1B). Eventually, because of mCa^2+^ influx, near complete depolarization of ΔΨ_m_ occurred in the pH 6.9 medium (Figure 1C), as shown by little change after adding CCCP, and by the complete depolarization of ΔΨ_m_ when Ca^2+^ recycling via the MCU was permitted (minus Ru360 group). Although adding CaCl_2_ at an external pH_e_ of 6.9 led eventually to near complete dissipation of ΔΨ_m_, when CsA was first added to the buffer, CsA prevented the gradual, slow extrusion of mCa^2+^ and declines in pH_m_ and ΔΨ_m_ induced by adding CaCl_2_ at pH_e_ 6.9 indicating a complete lack of CHE_m_ activity (see section “Supplementary Results S.2.1” and Supplementary Figures S.1A–C).

### Increasing Matrix Acidification Led to Ca^2+^ Efflux Until Loss of the ΔpH_m_ Gradient and a Lack of Ca^2+^ Re-uptake via MCU on Full Depolarization of ΔΨ_m_

A plot of extra-mitochondrial [Ca^2+^]_e_ as a function of matrix [H^+^]_m_ at each extra-mitochondrial pH (Figure 2) indicates maximal mCa^2+^ efflux occurred in the pH_e_ 6.9 medium (largest IMM (ΔH^+^] gradient), much less so in the pH 7.15 medium, and not at all in the pH 7.6 medium. Ca^2+^ efflux was accentuated in the presence of Ru360 given just after the added CaCl_2_ bolus (Figure 2). The difference (arrow) between the absence and presence of Ru360 indicates the rapid reuptake (recycling) of Ca^2+^ via MCU on extrusion via CHE_m_. Thus total Ca^2+^ efflux was greater in the pH 6.9 group when MCU was not blocked because [H^+^]_m_ rose higher than when MCU was blocked. The steep, vertical increase in mCa^2+^ efflux at the highest [H^+^]_m_ in the pH 6.9 group resulted from cessation of mCa^2+^ reuptake via MCU due to depolarization of ΔΨ_m_ (Figure 1C). The net amount of H^+^ entering mitochondria per Ca^2+^ exiting mitochondria may be indeterminate because much of the H^+^ entering is pumped out via the respiratory enzyme complexes.

### Mitochondrial Membrane Potential (ΔΨ_m_) Was Depressed by DNP After Adding CaCl_2_

In the protonophore series of experiments (time line, Figure 3), DNP alone decreased ΔΨ_m_ slightly as assessed by rodamine-123 (R123) (Huang et al., 2007) (Figure 4), in a concentration-dependent manner, except at 100 μM DNP, which alone fully (+OMN) or nearly (-OMN) depolarized ΔΨ_m_. ΔΨ_m_ was estimated as % of maximal depolarization, where the baseline after adding substrate with OMN signifies full polarization (0%) and addition of CCCP denotes complete depolarization (100%). Adding 10 μL of 0.1% DMSO (DNP vehicle) or 10 μM DNP had no significant effect when given before CaCl_2_, whereas adding 20, 30, or 100 μM DNP before 10 μM CaCl_2_ reduced the R123 ΔΨ_m_ signals by 12.7, 18.7, and 92.4% vs. DMSO (Figure 4A), respectively. In the presence of OMN (Figure 4C), adding 20, 30, or 100 μM DNP before 10 μM CaCl_2_ increased the fluorescence signal intensities (i.e., depolarized ΔΨ_m_) by 16.2, 33.0, and 99.0%, respectively, vs. DMSO (0%). Overall, before adding either 10 or 25 μM CaCl_2_, 20 and 30 μM DNP moderately decreased ΔΨ_m_ in the absence of OMN but greatly decreased ΔΨ_m_ in the presence of OMN, suggesting blocked proton pumping from complex V (Figure 4C,D vs. Figure 4A,B). If no CaCl_2_ was given after DNP, the moderate decrease in ΔΨ_m_, which was unaffected by CsA, persisted for up to 25 min (see section “Supplementary Results S.2.5” and Supplementary Figure S.7A). After adding 10 and 30 μM DNP, and then CaCl_2_, there were large decreases in ΔΨ_m_ resulting from entry of Ca^2+^. Although ΔΨ_m_ depolarization by DNP alone was unaffected by CsA, the subsequent slow ΔΨ_m_ depolarization induced by 25 μM CaCl_2_ was delayed by CsA (Supplementary Figure S.7B). Supplementary Results S.2.3 and Supplementary Figures S.3A–D shows statistics on mean ± SEM data for ΔΨ_m_ replotted from Figure 4 at time points 215, 275, and 700 s.

### Matrix Free [Ca^2+^]_m_ Rose or Fell Slowly Depending on Block of Complex V

**FIGURE 5:**
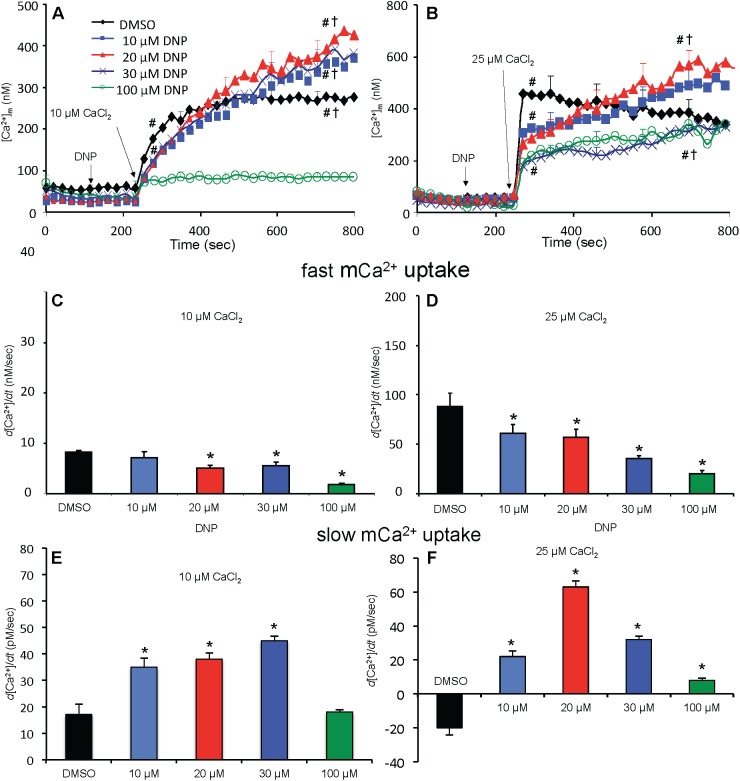
Change in [Ca^2+^]_m_ as a function of time **(A,B)** after adding DNP and CaCl_2_ in the absence of OMN. Adding DNP did not alter [Ca^2+^]_m_, *per se*, but did affect [Ca^2+^]_m_ depending on [DNP] and the amount of CaCl_2_ added in the absence of OMN. Adding 10 μM CaCl_2_ **(A)** caused a smaller increase in [Ca^2+^]_m_ than 25 μM CaCl_2_ **(B)**. In the absence of OMN the effect of DNP on [Ca^2+^]_m_ was less concentration-dependent and [Ca^2+^]_m_ continued to increase over time. Initial, rapid rates (averaged over 7 s) of increases in [Ca^2+^]_m_ (nM/s) as a function of [DNP] **(C,D)** just after adding CaCl_2_ in the absence of OMN. The rate of increase in [Ca^2+^]_m_ decreased as the degree of ΔΨ_m_ depolarization increased with increasing [DNP]. Note different Y-axis scales for 10 and 25 μM CaCl_2_. See Figure 4 for statistical notation for **(A–D)** plots. Much slower rates of increase in [Ca^2+^]_m_ (pM/s) occurred over time (slopes of data between 300 and 750 s) after the initial CaCl_2_ bolus **(E,F)**; the additional slow mCa^2+^ uptake was also dependent on ΔΨ_m_. Buffer pH = 7.15. Data obtained from seven hearts with 3–4 replicates per heart. For plots **(E,F)**, *P* < 0.05: ^∗^DNP vs. DMSO.

Adding 10 μM CaCl_2_ without DNP (ΔΨ_m_ fully polarized) caused [Ca^2+^]_m_ to increase rapidly from 80 nM (no added CaCl_2_) initially to 235 nM at 300 s, whereas after adding 25 μM CaCl_2_, [Ca^2+^]_m_ rose more rapidly to 450 nM (Figure 5A,B); [Ca^2+^]_m_ remained unchanged over time (300–750 s) after adding 10 μM CaCl_2_ but fell slightly and gradually (non-significantly) over time after adding 25 μM CaCl_2_ (DMSO group, Figure 5A,B). After adding 10*–*30 μM DNP, adding 10 μM CaCl_2_ promoted a slow, secondary rise in [Ca^2+^]_m_ (Figure 5A). The secondary, slow increase in [Ca^2+^]_m_ beginning 300 s after adding 10 μM CaCl_2_ plus DNP was accompanied by a slow decrease in extra-mitochondrial [Ca^2+^]_e_ (see Supplementary Figure S.6A). When ΔΨ_m_ was nearly or totally depolarized by 100 μM DNP in the absence of OMN, and after adding 10 μM CaCl_2_, there was no change in [Ca^2+^]_m_ over 300–750 s and thus no mCa^2+^ uptake over time (Figure 5A). [Ca^2+^]_m_ slowly increased over 300–750 s after first adding 10 and 20 μM DNP and then 25 μM CaCl_2_ (Figure 5B), which caused the slow declines in ΔΨ_m_ (Figure 4B). In the 100 μM DNP group [Ca^2+^]_m_ increased moderately immediately after adding 25 μM CaCl_2_, but did not change further over time. Supplementary Results S.2.6 and Supplementary Figures S.4A,B display statistics on mean ± SEM data for [Ca^2+^]_m_ replotted from Figure 5 (-OMN) at time points 215, 275, and 700 s.

**FIGURE 6:**
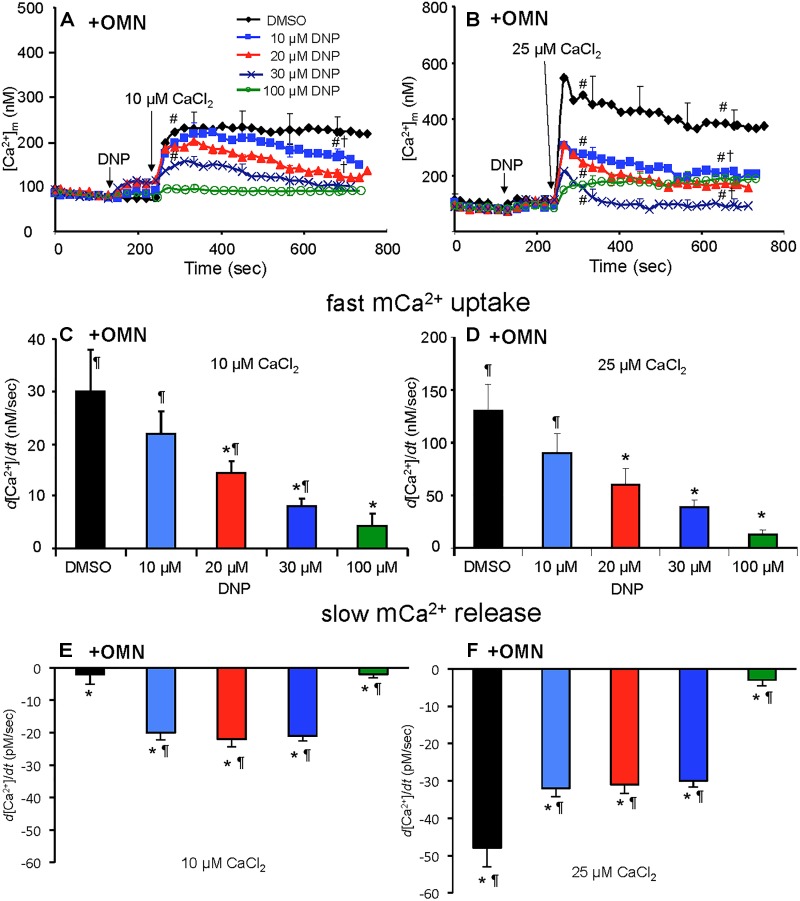
Change in [Ca^2+^]_m_ as a function of time **(A,B)** after adding DNP and CaCl_2_ in the presence of OMN. Adding DNP did not alter [Ca^2+^]_m_, *per se*, but did affect [Ca^2+^]_m_ depending on [DNP], the amount of CaCl_2_ added, and the presence of OMN. Adding 10 μM CaCl_2_ **(A)** caused a smaller increase in [Ca^2+^]_m_ than 25 μM CaCl_2_ **(B)**. In the presence of OMN, DNP caused concentration-dependent decreases in [Ca^2+^]_m_ over time. Initial, rapid rates (averaged over 7 s) of increase in [Ca^2+^]_m_ (nM/s) as a function of [DNP] **(C,D)** just after adding CaCl_2_ in the presence of OMN. The rate of increases in [Ca^2+^]_m_ decreased as the degree of ΔΨ_m_ depolarization increased with increasing [DNP]. See Figure 4 for statistical notation for **(A–D)** plots. A slow rate of decrease in [Ca^2+^]_m_ (pM/s) occurred over time (slopes of data between 300 and 750 s) after the initial CaCl_2_ bolus **(E,F)**; the slow mCa^2+^ efflux was dependent on slow mCa^2+^ influx (hidden by OMN treatment) (see Figure 6 vs. Figure 5) and a slow fall in matrix pH and ΔΨ_m_. Note different Y-axis scales for 10 and 25 μM CaCl_2_. Buffer pH = 7.15. Data obtained from seven hearts with 3–4 replicates per heart. For plots **(E,F)**, *P* < 0.05: ^∗^DNP vs. DMSO. ^¶^+OMN vs. —OMN (Figure 5) for same [DNP].

In marked contrast, when complex V was blocked by OMN, adding 10 μM CaCl_2_ (Figure 6A) after adding10–30 μM DNP caused a marked decrease in [Ca^2+^]_m_ over time (300–750 s); after adding 25 μM CaCl_2_ in the absence of DNP (Figure 6B), [Ca^2+^]_m_ rose higher initially, whereas 10–30 μM DNP caused a slow decrease in [Ca^2+^]_m_ over this period, indicating net mCa^2+^ efflux. Supplementary Results S.2.6 and Supplementary Figures S.4C,D shows statistics on mean ± SEM data for [Ca^2+^]_m_ replotted from Figure 6 (+OMN) at time points 215, 275, and 700 s. The secondary, slow decrease in [Ca^2+^]_m_ after adding 20 μM DNP plus 25 μM CaCl_2_ was accompanied by an increase in extra-mitochondrial [Ca^2+^]_e_ (see Supplementary Figure S.6B). Note that additional mCa^2+^ uptake after giving 25 μM CaCl_2_ was halted after adding Ru360, 90 s later (at *t* = 325 s) and converted to mCa^2+^ efflux in the presence of OMN as shown by the increase in [Ca^2+^]_e_ (see Supplementary Figures S.6B vs. S.6A).

A summary of slope data collected over the first 7 s (1 sample/s) after adding 10 or 25 μM CaCl_2_ in the absence (Figure 5C,D) or presence (Figure 6C,D) of OMN shows that the average initial, rapid increase in [Ca^2+^]_m_ via the MCU was much faster after adding 25 μM CaCl_2_ than after 10 μM CaCl_2_ in the ± OMN groups; this initial rate of mCa^2+^ uptake decreased as ΔΨ_m_ fell with added DNP. The initial rate of increase in [Ca^2+^]_m_ during the first 7 s after adding 10 μM CaCl_2_ (Figure 5C) decreased from 8 to 2 nM/s (DNP 0–100 μM). After adding 25 μM CaCl_2_ (Figure 5D), the rate decreased from 88 to 20 nM/s. In the presence of OMN (Figure 6C,D), the initial increases in [Ca^2+^]_m_ in fully coupled mitochondria (no DNP) were larger than those in the absence of OMN (Figure 6C,D vs. Figure 5C,D). With OMN present, the initial increases in [Ca^2+^]_m_ decreased from 30 to 4 nM/s after adding 10 μM CaCl_2_ and from 130 to 13 nM/s after adding 25 μM CaCl_2_, Thus the initial rates of increase in [Ca^2+^]_m_ with 10 μM CaCl_2_ were consistently faster in the presence of OMN (Figure 6C vs. Figure 5C), and at 25 μM CaCl_2_, with or without 10 μM DNP (Figure 6D vs. Figure 5D).

A summary of slope data collected between 300 and 750 s, i.e., after the initial, rapid increase in [Ca^2+^]_m_ via the MCU with added 10 μM CaCl_2_, demonstrates a much slower and smaller (pM/s) gradual increase in [Ca^2+^]_m_ over time in the absence of OMN with a threefold greater slope after 30 μM DNP vs. DMSO (Figure 5E). After adding 25 μM CaCl_2_, the slow increase in [Ca^2+^]_m_ was about fourfold higher after 20 μM DNP vs. DMSO (Figure 5F). The secondary slow rise in [Ca^2+^]_m_ was about 1000 times slower than the initial fast phase and roughly dependent on both the amount of mCa^2+^ that was taken up initially just after adding CaCl_2_ and the extent of ΔΨ_m_ depolarization. In contrast, in the presence of OMN under the same conditions of added CaCl_2_ and DNP, the slope data showed slow and small declines (rather than increases) in [Ca^2+^]_m_ over time (Figure 6E,F). The slow rate of extrusion of mCa^2+^ by CHE_m_ when complex V was blocked with OMN (Figure 6E,F) became greater when mCa^2+^ entry via the MCU was greater (Figure 6 A,B).

### Matrix pH Remained Steady Without OMN but Fell With OMN-Induced Block of Complex V

**FIGURE 7:**
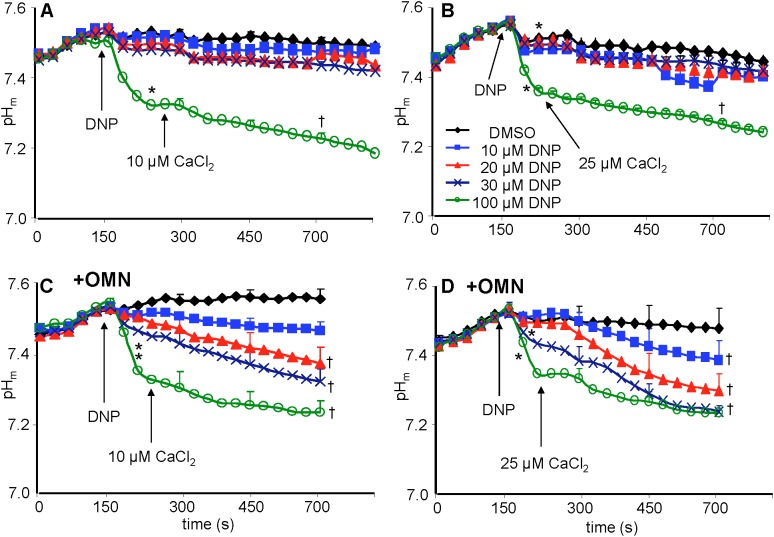
Change in pH_m_, measured with BCECF, as a function of time after adding DNP and CaCl_2_ in the absence **(A,B)** or presence **(C,D)** of OMN. Note that except for 100 μM DNP, neither DNP nor added CaCl_2_ altered pH_m_ in the absence of OMN **(A,B).** However, in the presence of OMN **(C,D)** graded decreases of ΔΨ_m_ by DNP caused a graded increase in matrix acidity that was further increased in the presence of added CaCl_2_ **(C,D)**. Buffer pH = 7.15. Data obtained from 10 hearts with 3–4 replicates per heart. For *P* < 0.05: ^∗^after DNP vs. before DNP; ^†^late (700 s) vs. early (215 s) after adding CaCl_2_.

Baseline matrix pH_m_ was approximately 7.55 in each group after adding PA and before adding DNP (Figure 7A–D). In the absence of OMN, adding 10–30 μM DNP did not result in a significant net decrease in pH_m_; however, 100 μM DNP markedly decreased pH_m_ (Figure 7A,B). This effect to collapse the ΔpH_m_ gradient was proportional to the collapse of the ΔΨ_m_ gradient (Figure 4). In the absence of OMN, adding CaCl_2_ had no appreciable effect on pH_m_ (ΔΨ_m_ partially depolarized) even in the presence of DNP, except for 100 μM DNP, when pH_m_ fell markedly (ΔΨ_m_ fully depolarized) (Figure 7A,B). In the absence of OMN, H^+^ influx was matched by H^+^ pumping as pH_m_ did not change appreciably. In contrast, in the presence of OMN there was a strong DNP concentration-dependent fall in matrix pH_m_ (Figure 7C,D) after adding CaCl_2_. This fall in pH_m_ was likely due to blocked H^+^ pumping by complex V in the presence of OMN (see below). Supplementary Figures S.5A–D shows statistics on mean ± SEM data on pH_m_ replotted from Figure 7 (main text) at time points 215, 275, and 700 s. Supplementary Figure S.8 displays plots of pH_m_ as a function of [Ca^2+^]_m_ at 700 s after adding DNP and CaCl_2_; these correlations show how [Ca^2+^]_m_ decreases while pH_m_ decreases in the presence, but not in the absence of OMN.

### Mitochondrial Redox State Remained Steady Without OMN but Fell With OMN-Induced Block of Complex V

**FIGURE 8:**
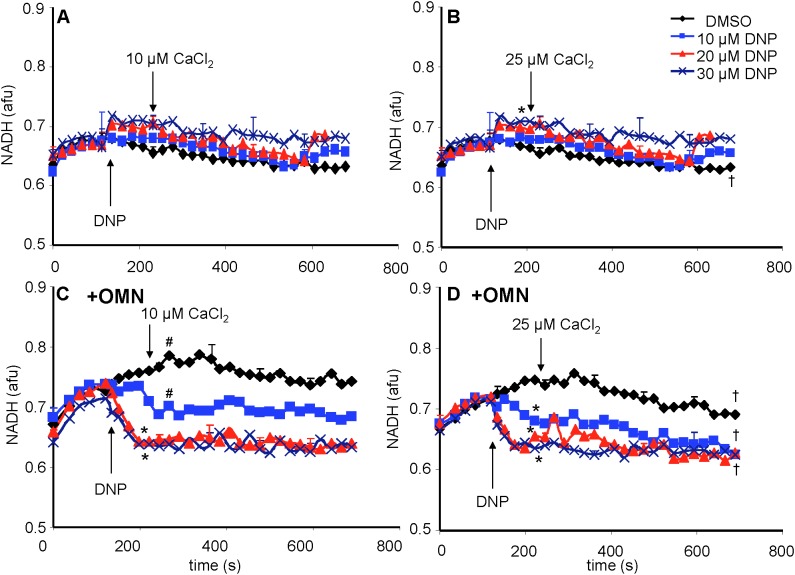
Change in mitochondrial redox state, measured by NADH autofluorescence, as a function of time after adding DNP and CaCl_2_ in the absence **(A,B)** or presence **(C,D)** of OMN. Note that the redox state was maintained after adding 10–30 μM DNP and CaCl_2_ in the absence of OMN **(A,B)** but that in the presence of OMN **(C,D)** there was a concentration-dependent decrease in NADH autofluorescence. Adding CaCl_2_ did not alter DNP-induced changes in redox state in the presence of OMN. Buffer pH = 7.15. Data obtained from eight hearts with 3–4 replicates per heart. See Figure 4 for statistical notation.

A reduced redox state is associated with maintenance of pH_m_. Adding the substrate PA increased the redox state (more reduced) as determined by high NADH autofluorescence (Figure 8). In the absence of OMN, adding 10 to 30 μM DNP ± 10 or 25 μM CaCl_2_ (Figure 8A,B) did not cause a significant change in NADH. NADH was unchanged despite up to 60% decrease in ΔΨ_m_ fluorescence (Figure 4A,B) after adding DNP and CaCl_2_. However, when complex V was blocked by OMN (Figure 8C,D), there was significant oxidation (low NADH) by DNP in a concentration dependent manner. In contrast to the condition without OMN, with OMN present as little as a 20% fall in ΔΨ_m_ fluorescence (Figure 4C,D) led to a more oxidized NADH state. Moreover, NADH was fully oxidized at 20 μM DNP with OMN present (Figure 8C,D), and the oxidized state was not altered significantly by adding CaCl_2_ after DNP. In the absence or presence of CaCl_2_, NADH was completely oxidized after adding 100 μM DNP (data not shown).

### ATP Concentration Fell Without OMN but Remained Steady With OMN-Induced Block of Complex V

**FIGURE 9:**
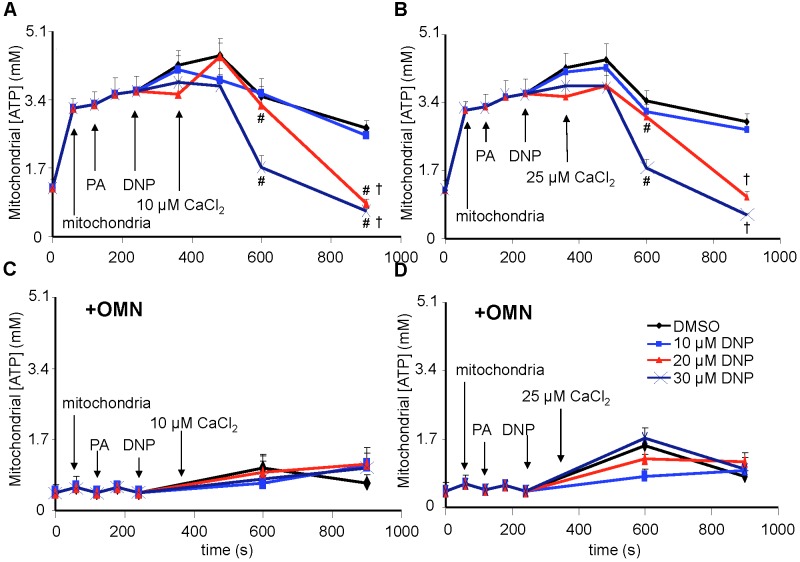
[ATP], measured in total solution by the luciferin-luciferase reaction, and expressed as the calculated mitochondrial [ATP], was altered as a function of DNP and added CaCl_2_ in the absence **(A,B)** and presence **(C,D)** of OMN. Adding mitochondria to the respiration buffer (first arrow) increased [ATP]; adding pyruvic acid (PA) and DNP had no additional effect; but adding CaCl_2_ after DNP in the absence of OMN resulted in concentration-dependent decreases in [ATP] as graded depolarization of the IMM occurred (Figure 4). F_O_F_1_-ATP synthase/ase activity was near zero and remained essentially unchanged after adding PA, DNP, and CaCl_2_ in the presence of OMN **(C,D)**. Note that [ATP] was not measured continuously, so the lines between sampling points do not represent averaged data for any given intermediate time period between points. ADP/ATP ratio results are also given (see section “Supplementary Materials S.1.1, S.1.12 and Supplementary Results S.2.9”). Data obtained from 20 hearts. See Figure 4 for statistical notation.

Total medium [ATP] was measured and mitochondrial [ATP]_m_ was estimated (see section “Supplementary Materials S.1.10”). Basal [ATP]_m_ was measured after adding mitochondria to the experimental buffer in the absence of OMN (Figure 9A,B). There was no change in basal [ATP]_m_ after adding PA. DNP, at 10 μM, did not significantly change [ATP] before or after adding CaCl_2_ (Figure 9A,B). Basal [ATP]_m_ was unchanged if CaCl_2_ was not added (data not displayed). Adding 20 or 30 μM DNP alone had no significant effect on [ATP]_m_, but adding CaCl_2_ resulted in a decrease in [ATP]_m_ (Figure 9A,B). In the presence of OMN (Figure 9C,D), adding mitochondria to the buffer did not change [ATP]_m_, indicating inhibited complex V activity. [ATP]_m_ remained at a very low level and was unaffected by DNP or CaCl_2_ in the presence of OMN. With OMN present, ATP_m_/ADP_m_ ratios (see section “Supplementary Materials S.1.11, S.1.12 and Supplemental Results S.2.9”) also decreased with added DNP and CaCl_2_, along with the progressive declines in ΔΨ_m_.

### Additional Supplemental Comparisons and Calculations

Supplementary Results S.2.2 and Supplementary Figure S.2 demonstrate the effect of adding DNP and CaCl_2_ on respiration. Supplementary Results S.2.7 and Supplementary Figure S.9 furnish values for ΔΨ_m_, [Ca^2+^]_m_, and pH_m_ at 700 s, replotted from Figure 4–7, to compare these results in the presence or absence of OMN. The Supplementary Table shows DNP concentrations that produced 50% inhibitions (IC_50_) of ΔΨ_m_, [Ca^2+^]_m_, fast (initial) *d*[Ca^2+^]_m_/*dt*, and pH_m_ as a linear function of 0*–*30 μM DNP ± OMN at the 700 s time point. Supplementary Figure S.10 displays calculated mCa^2+^ flux rates (*J*_CHE_) for CHE_m_ (see section “Supplementary Results S.2.8”) in the absence and presence of OMN.

## Discussion

### Ca^2+/^H^+^ Exchange Activity Is Identified by Manipulating IMM Δ[H^+^] and Δ[Ca^2+^] Gradients

**FIGURE 10:**
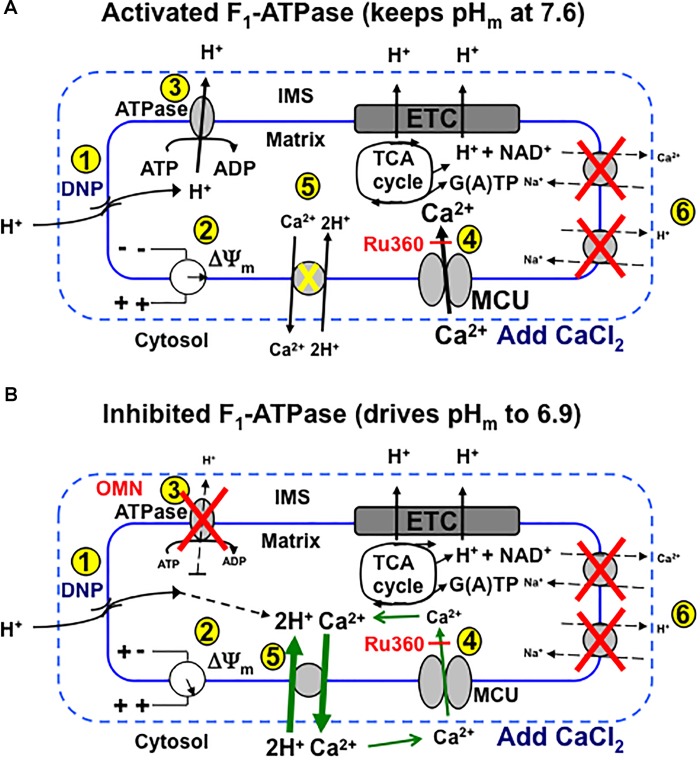
Schema depicting putative role of MCU and CHE_m_ on slow Ca^2+^ influx and efflux, respectively, during stepwise depolarization with DNP with un-inhibited (i.e., minus OMN) **(A)** vs. inhibited **(B)** F_O_F_1_-ATPsynthase(ase) (i.e., plus OMN) after a bolus addition of CaCl_2_ to the mitochondrial medium. **(A)** (1) DNP permits H^+^ entry that tends to (2) decrease ΔΨ_m_, which enhances H^+^ pumping by respiratory complexes, including (3) F_O_F_1_-ATPase, so that pH_m_ does not decrease appreciably and ΔΨ_m_ is partially supported (2). Adding CaCl_2_ further depolarizes ΔΨ_m_ by allowing more cationic (Ca^2+^) charges into the matrix via the MCU (4). Over time, in the range of a 20–60% decline in ΔΨ_m_, pH_m_ remains unchanged as (5) H^+^ is pumped out (3) in exchange for permissive H^+^ entry (1) in triggering additional slow mCa^2+^ uptake by MCU and causing ΔΨ_m_ to decrease further. CHE_m_ is inhibited by the lack (pH 7.6) of matrix acidity (5) and NCE_m_ and NHE_m_ are inactivated by the lack of substrate and buffer Na^+^ (6). **(B)** Alternatively, when F_O_F_1_-ATPase is inhibited (3), matrix acidity gradually increases (pH 6.9), ΔΨ_m_ is less supported (2) and Ca^2+^ slowly exits (CHE_m_) in exchange for slow H^+^ entry due to DNP (5). This sequence triggers a net loss of mCa^2+^ even though uptake of Ca^2+^ via the MCU continues, as shown by a greater efflux of mCa^2+^ by CHE_m_ when additional mCa^2+^ uptake via MCU is blocked by Ru360 (4). DNP, dinitrophenol; ETC, electron transport chain; IMS, inner membrane space; MCU, mitochondrial Ca^2+^ uniporter; OMN, oligomycin; TCA, tricarboxylic acid.

We provide firm support for a role of CHE_m_ in maintaining homeostasis of Ca^2+^ against H^+^ under certain conditions in cardiac cell mitochondria that may mimic some sequelae of cardiac IR injury. Our results: (1) furnish direct evidence for CHE_m_ activity by the secondary, slow increases in matrix Ca^2+^ efflux coupled to slow increases in matrix H^+^ influx, when both NCE and NHE activities are blocked, and particularly, when MCU-dependent mCa^2+^ re-uptake is blocked with Ru360; (2) demonstrate that respiration increases while ΔΨ_m_ decreases mildly, whereas pH_m_ and redox state are relatively maintained when inducing a matrix inward H^+^ leak with DNP before adding CaCl_2_; adding CaCl_2_ results in a secondary, slow increase in [Ca^2+^]_m_ that slowly depolarizes ΔΨ_m_; (3) show that with permissive H^+^ influx, but inhibited outward H^+^ pumping at complex V, adding CaCl_2_ causes larger decreases in ΔΨ_m_, pH_m_, and NADH and results in a slow decrease in [Ca^2+^]_m_; (4) indicate that blocking complex V with OMN to prevent H^+^ pumping causes ΔΨ_m_ to further decrease after adding CaCl_2_ because the influx of mCa^2+^ via the MCU is not opposed by H^+^ pumping at complex V; (5) suggest that the lack of a slow fall or rise in [Ca^2+^]_m_ in the presence of 100 μM DNP is due to the loss of ΔΨ_m_-dependent mCa^2+^ uptake by MCU; (6) point out that only in partially depolarized mitochondria does added CaCl_2_ result in a pH_m_-independent gradual increase in [Ca^2+^]_m_ that is reciprocated by H^+^ pumping to maintain pH_m_; preventing matrix acidification is associated with a maintained redox state; and (7) show that the decrease in [ATP] in the absence of OMN supports ATP hydrolysis with H^+^ pumping. These two scenarios, ±OMN, are depicted graphically in Figure 10A vs. Figure 10B.

### Net Mitochondrial Ca^2+^ Influx Occurs via MCU and Net Ca^2+^ Efflux Can Occur via Ca^2+^/H^+^ Exchange

The dependence of rapid MCU-mediated mCa^2+^ uptake on ΔΨ_m_ has been examined extensively (Gunter and Pfeiffer, 1990; Gunter et al., 1994; Dash et al., 2009; Haumann et al., 2010). But our study demonstrates that net m[Ca^2+^] can additionally increase slowly via the MCU, and that this happens when pH_m_ is relatively maintained despite a decline in ΔΨ_m_ resulting from the DNP-mediated inward H^+^ flux and after the initial rapid Ca^2+^ influx via MCU. A gradual increase in [Ca^2+^]_m_ at the expense of maintaining the ΔpH_m_ may be deleterious to mitochondrial function. We propose that this secondary rise in net [Ca^2+^]_m_ results from an adequate ΔΨ_m_ with Ru360-dependent slow mCa^2+^ influx, which eventually leads to a slow, continued fall in ΔΨ_m_. Because H^+^ pumping at complex V maintains the Δ[H^+^]_m_ gradient, mCa^2+^ efflux via CHE_m_ in exchange for mH^+^ influx due to the H^+^ leak is likely masked by mCa^2+^ re-uptake. Thus, the DNP-induced H^+^ leak and the concomitant dissipation of the IMM Δ[H^+^] gradient, when countered by H^+^ pumping at complex V (in addition to other complexes), can maintain the ΔpH_m_ and support the *pmf* (ΔΨ_m_ + RT/FΔpH_m_) (Dzbek and Korzeniewski, 2008). This view is especially supported by the smaller decline in extra-mitochondrial [Ca^2+^]_e_ in the presence of 20 μM DNP, 25 μM CaCl_2_, and OMN, as well as in the presence of Ru360, by the gradual increase in [Ca^2+^]_e_ due to CHE_m_ mediated Ca^2+^ efflux. These results are reinforced by the exaggerated effect of added CaCl_2_ to enhance the decline in ΔΨ_m_ over time and by the slow decreases in [Ca^2+^]_m_ linked to slow decreases in pH_m_. Blocking outward H^+^ pumping by complex V prevented compensation for DNP-mediated H^+^ influx. Consistent with our observations, it was reported that matrix acidification may reduce Ca^2+^ uptake in cardiac mitochondria by its effect on decreasing ΔΨ_m_ (Gursahani and Schaefer, 2004). In contrast, when ATP_m_ hydrolysis is prevented, pH_m_ slowly decreases toward pH_e_ with a greater fall in ΔΨ_m_; the slow H^+^ influx is accompanied by a slow net fall in [Ca^2+^]_m_ mediated by CHE_m_ even though the extruded Ca^2+^ is recycled via the MCU. Since H^+^ influx (DNP-induced leak) is not countered by reciprocal H^+^ pumping to restore ΔpH_m_, the slow influx of H^+^ is exchanged for slow Ca^2+^ efflux via CHE_m_ until the ΔpH gradient is dissipated.

Ca^2+^ and H^+^ gradients across the IMM are largely dependent on ΔΨ_m_ and ΔpH gradients resulting from H^+^ pumping by respiratory complexes. Ionic homeostasis requires one cation efflux pathway to oppose another cation influx pathway and *vice versa*. Cation exchangers fulfill this need. Unlike mCa^2+^ uptake via MCU, which is dependent on ΔΨ_m_ and on the chemical gradient, exchange of Ca^2+^ and H^+^ via CHE_m_ may or may not be dependent on ΔΨ_m_ (Rottenberg and Marbach, 1990; Gunter et al., 1991). But the direction of Ca^2+^ and H^+^ flux mediated solely by CHE_m_ is dependent on a large IMM [H^+^] or [Ca^2+^] gradient to shuttle Ca^2+^ or H^+^ across the IMM. This can be expressed by an electroneutral *J*_CHE_ flux equation (Tewari et al., 2014), calculated here in the presence and absence of OMN (see section “Supplementary Results S.2.8” and Supplementary Figure S.10). *J*_CHE_ flux analysis of our data suggests that slow mCa^2+^ influx could have occurred via CHE_m_ in the absence of OMN, whereas mCa^2+^ efflux could have occurred in the presence of OMN. Indeed, we have provided strong support for slow net mCa^2+^ efflux mediated by CHE_m_ (despite slow mCa^2+^ uptake by MCU) when complex V cannot pump H^+^ in the presence of OMN.

Although CHE_m_ likely occurs both in the absence or presence of OMN, our results suggest that the observed secondary, slow influx of mCa^2+^ influx (minus OMN) is due primarily to re-uptake by a Ru360 sensitive mechanism, presumably MCU, that may overwhelm any CHE_m_ activity. This is because Ru360 blocked the slow rise in [Ca^2+^]_m_ and the slow fall in [Ca^2+^]_e_, thus supporting MCU as the mediator of the slow mCa^2+^ influx. The *J*_CHE_ flux equation only monitors differences in [H^+^] and [Ca^2+^] on either side of the IMM and does not rely on effects of the ΔpH_m_ gradient on H^+^ pumping or the ΔΨ_m_ gradient on mCa^2+^ uptake via MCU. Thus the secondary, slow mCa^2+^ uptake after the initial CaCl_2_ bolus (Figure 5A,B,E,F) appears to be a direct effect of H^+^ pumping by complex V (minus OMN) to maintain the ΔpH_m_ charge gradient and support the *pmf* although ΔΨ_m_ continues to fall due to the continued mCa^2+^ influx. On the other hand, inhibiting ATP_m_ hydrolysis (Figure 9C,D) to prevent H^+^ pumping not only enhances the fall in ΔΨ_m_ (Figure 4C,D) to retard further mCa^2+^ loading by the MCU, but also permits slow CHE_m_-mediated mCa^2+^ efflux (Figure 6A,B,E,F) in exchange for mH^+^ influx until the diminishing ΔpH_m_ gradient is abolished (Figure 7C,D).

Alternatively, we demonstrated CHE_m_ activity by acidifying the external medium before adding CaCl_2_, while blocking NCE_m_ and NHE_m_ activities by using Na^+^ free buffer and substrates. We observed a slowly increasing [Ca^2+^]_e_ coupled to a slowly increasing [H^+^]_m_. We used Ru360 to expose the net amount of mCa^2+^ efflux via CHE_m_ by blocking the effluxed Ca^2+^ from re-entering via MCU (Figure 1, 2). It is unlikely that 0.1–1 μM Ru360 inhibits CHE_m_ because Ru360 did not block mCa^2+^ efflux (Figure 1, 2), only mCa^2+^ influx. Of course, Ru360 might block another mode of non-MCU Ca^2+^ uptake. Our proposed mechanism is described schematically in Figure 10A,B. We postulate that CHE_m_ activity is completely inhibited if the matrix remains alkaline (large ΔpH_m_ gradient), thus exposing net Ca^2+^ uptake via MCU. The slow increases in [Ca^2+^]_m_ that we observed previously (Haumann et al., 2010) likely represent net slow mCa^2+^ via MCU (reference Figure 5).

A leucine zipper EF hand-containing trans-membrane protein (LETM1) found in non-mammalian cells is thought to be a molecular component of CHE_m_ (Jiang et al., 2009; Shao et al., 2016). Knockdown and expression of LETM1 in a number of cell lines support its role in Ca^2+^/H^+^ exchange, particularly in mitochondria (Jiang et al., 2013; Doonan et al., 2014). Alternatively, other studies (Nowikovsky et al., 2004, 2012; Froschauer et al., 2005; Malli and Graier, 2010; Austin et al., 2017) support that LETM1 either does not mediate Ca^2+^ efflux (De Marchi et al., 2014) or that it mediates K^+^/H^+^ and/or Na^+^/H^+^ exchange, so conclusive genetic evidence for CHE requires more study. It is important to note that the elusive CHE protein appears to be insensitive to MCU inhibitors, i.e., ruthenium red (RR) compounds (Bernardi et al., 1984), and to CGP-37157, the NCE inhibitor (Tsai et al., 2014). The present study explores for the first time the kinetics of CHE_m_ activity in relation to MCU activity in cardiac cell mitochondria.

### ΔΨ_m_ < E_REV-ATPase_ Promotes ATP Hydrolysis

F_O_F_1_-ATPsynthase/ase directionality is governed by ΔΨ_m_ and its “reversal potential” *E*_REV -ATPase_, which in turn is dependent on the concentration of the reactants ATP/ADP, and H^+^ (Metelkin et al., 2009; Chinopoulos and Adam-Vizi, 2010; Chinopoulos et al., 2010). Additional factors of *E*_REV_ that affect the direction and rate of ATP synthesis/hydrolysis are the free [P_i_] and the H^+^_m_/ATP_m_ coupling ratio, *n* (Cross and Muller, 2004). When ΔΨ_m_ becomes less negative than *E*_REV_, which depends on a high [ATP]_m_ and ΔpH_m_, but a low [ADP]_m_, H^+^ ejection by complex V becomes thermodynamically favorable (Metelkin et al., 2009; Chinopoulos and Adam-Vizi, 2010; Chinopoulos et al., 2010; Chinopoulos, 2011). *E*_REV-ATPase_ can occur when ΔΨ_m_ falls between -130 and -100 mV, depending on matrix [ATP]_m_/[ADP]_m_, [P_i_]_m_, ΔpH_m_, and the coupling ratio (Chinopoulos et al., 2010; Chinopoulos, 2011). Others (Leyssens et al., 1996; Bains et al., 2006; Chinopoulos and Adam-Vizi, 2010) have observed that a fall in ΔΨ_m_ caused by a protonophore, such as DNP or CCCP, can induce ATP hydrolysis through reversal of F_O_F_1_-ATPsynthase. The consequent H^+^ pumping by complex V would tend to partially restore ΔΨ_m_ to offset the protonophore-induced decreases in pH_m_ and ΔΨ_m_ as discussed above. The electrical gradient ΔΨ_m_ and the H^+^ chemical gradient Δ[H^+^]_m_ together contribute to the total *pmf* that powers the synthesis of ATP; when *pmf* is not maintained, hydrolysis of matrix ATP occurs. Previous studies have also furnished indirect evidence for reversal of F_O_F_1_-ATPsynthase under conditions of reduced mCa^2+^ uptake and a fully depolarized ΔΨ_m_ with CCCP (Leyssens et al., 1996; Bains et al., 2006). ATP_m_ hydrolysis has been reported to occur *in vivo* during cardiac ischemia (Grover et al., 2004), but the *in vivo* ΔΨ_m_ at which this occurs is not known. Here we show how a DNP-induced fall in ΔΨ_m_ induces ATP hydrolysis.

In the absence of OMN, the lack of a fall in ATP levels after adding 10 μM DNP indicated that ATP_m_ hydrolysis (Figure 9) did not occur because ΔΨ_m_ remained relatively stable before adding CaCl_2_. However, adding CaCl_2_ resulted in a gradual, but large, fall in ΔΨ_m_ over time. In the presence of 20 μM DNP and 25 μM CaCl_2_, ATP hydrolysis occurred (20–25% of maximum) with a decrease in ΔΨ_m_ at an IMM gradient of approximately 0.35 ΔpH_m_ units (Figure 7A,B). A faster rate of ATP hydrolysis was indicated by the additional fall in [ATP]_m_ over time after adding 30 μM DNP and CaCl_2_. The DNP-induced falls in ΔΨ_m_ were accompanied by reduced ATP_m_/ADP_m_ ratios (see section “Supplementary Materials S.1.11, S1.12 and Supplementary Results S.2.9”) indicating consumption of ATP, as also shown by the lower [ATP]_m_ (Figure 9A,B). A calculation of available matrix ATP is given (see section “Supplementary Results S.2.10”). In the presence of 100 μM DNP and added CaCl_2_, ΔΨ_m_ was maximally depolarized (Figure 4A,B), the ΔpH_m_ gradient was abolished (Figure 7A,B), and NADH was oxidized (Figure 8A,B), indicating that ATP_m_ hydrolysis was insufficient to maintain the *pmf*. This contrasts to the situation with 10–30 μM DNP where *pmf* was supported largely by the ΔpH_m_ gradient, as also reflected by the maintained NADH redox state.

ΔΨ_m_ is normally fully polarized when complex V is blocked by OMN (Valdez et al., 2006; Brand and Nicholls, 2011); however, the effect of DNP to slightly decrease ΔΨ_m_ was intensified when OMN was present, particularly after adding 25 μM CaCl_2_ that intensifies the depolarization of ΔΨ_m_ in the presence of DNP. This effect of DNP in the absence of OMN indicates that ATP hydrolysis indeed supported the ΔpH_m_ via H^+^ pumping even at a relatively small decline in ΔΨ_m_ with DNP. With OMN present, ATP hydrolysis cannot occur (Figure 9C,D) and so complex V cannot contribute to maintaining pH_m_; therefore, the low pH_m_ accompanied by a high [Ca^2+^]_m_ must have activated CHE_m_.

### Changes in pH_m_, [Ca^2+^]_m_, and NADH Are Larger With OMN Than Without OMN

An interesting observation of our study is the contribution of complex V to maintain the ΔpH_m_ gradient (and thus supporting the *pmf*) whereby the H^+^ leak is compensated by augmented H^+^ pumping by complex V; this resulted in slow mCa^2+^ influx (“Ca^2+^ leak”) that could be blocked by Ru360, which indicates the influx likely occurred via MCU. But if compensatory H^+^ pumping is blocked by OMN, the matrix becomes acidic, the ΔpH_m_ gradient falls lower, and slow mCa^2+^ efflux occurs via CHE_m_ thus masking the slow mCa^2+^ influx (Figure 10B). Evidence for H^+^ pumping during ATP hydrolysis during DNP-mediated H^+^ influx was provided by the maintenance of an alkaline pH_m_; moreover, pH_m_ indeed fell when H^+^ pumping was blocked by OMN. Similarly, if mitochondria reside in an acidic environment (Figure 1, 2), [H^+^]_m_ falls as [Ca^2+^]_e_ rises, indicating CHE_m_. Indeed, in a previous study it was reported that adding lactic acid to a Na^+^ free mitochondrial suspension increased buffer Ca^2+^ by 43% (Gambassi et al., 1993); it was suggested that Ca^2+^ was extruded as H^+^ influx caused H^+^ ions to compete with Ca^2+^ ions for mitochondrial binding sites (Gambassi et al., 1993). We furnish direct evidence for a link between Ca^2+^ efflux with H^+^ influx in mammalian cardiac muscle mitochondria, when Na^+^ is absent and the MCU is blocked after adding CaCl_2_.

NADH levels remained unchanged after adding DNP and CaCl_2_ (Figure 8A,B); this likely reflects the faster state 2 respiration (Supplementary Figure S2) since the inward H^+^ leak by DNP was balanced by H^+^ pumping from complex V as well as from complexes I, III, and IV. Only at 100 μM DNP with CaCl_2_, which fully depolarized ΔΨ_m_ (Figure 4A,B), did DNP result in a lower pH_m_ (Figure 7A,B) and a more oxidized redox state, i.e., a decrease in NADH (Figure 8A,B). It is likely that an increase in F_O_F_1_-ATPase activity plus a faster TCA cycle turnover (increased NADH/NAD^+^ ratio) can result in maintained NADH levels despite the DNP-induced H^+^ leak. In the presence of OMN, however, NADH was gradually oxidized (Figure 8C,D) along with the fall in pH_m_ (Figure 7C,D); this scenario likely occurred because the additional H^+^ pumping by complex V to support ΔΨ_m_ was blocked. We observed that adding CaCl_2_ alone did not significantly change NADH levels in this model, which is consistent with our earlier study (Haumann et al., 2010). Although an increase in [Ca^2+^]_m_ can stimulate NADH producing dehydrogenases (Denton et al., 1980; McCormack and Denton, 1980; Wan et al., 1989; Brandes and Bers, 1997), our experiments were conducted at maximal [Ca^2+^]_m_ values below the *K_0.5_* of 1 μM Ca^2+^ at which these dehydrogenases are reported to be activated (Denton et al., 1980; McCormack and Denton, 1980).

### What Is the Functional Role of CHE_m_: How Is Net mCa^2+^ Efflux Modified by mCa^2+^ Influx via MCU?

The net Ca^2+^ driving force for ions across the IMM can be estimated by Nernst equilibrium potentials for given estimates of ΔΨ_m_. Under conditions of 20 μM DNP, 25 μM CaCl_2_, and in the absence of OMN, when [Ca^2+^]_m_ slowly increased, we calculated Nernst equilibrium potentials of approximately -8 and +18 mV, respectively, for [Ca^2+^] and [H^+^] at 700 s. We estimated ΔΨ_m_ as -110 to -120 mV at 700 s (based on our values for % of minimal and maximal depolarization (R-123 fluorescence) and curve fitting for approximating conversion to ΔΨ_m_ (Huang et al., 2007)). This indicated that the driving force for both Ca^2+^ and H^+^ would remain inward despite H^+^ pumping at complex V to attempt to re-establish the ΔpH_m_ gradient by compensating for the DNP-mediated H^+^ influx. Based on our estimated ΔΨ_m_ and the calculated Ca^2+^ and H^+^ equilibrium potentials driving both Ca^2+^ and H^+^ inward, we conclude that the outward H^+^ pumping by complex V (in addition to complexes I, III, IV) was sufficient to compensate for the continued inward influx of H^+^ mediated by DNP thus restoring the ΔpH_m_ gradient, but not the *pmf*, and thus preventing activation of CHE_m_. Ru360 blocked this additional uptake of mCa^2+^ by the MCU so that [Ca^2+^]_e_ did not continue to fall.

We predict that the major conduit for both fast and slow mCa^2+^ influx under our experimental conditions occurs primarily via the MCU. The efflux of Ca^2+^ via the CHE_m_ is slow so we expect the re-uptake of Ca^2+^ via the MCU also would be slow. Although the *J*_CHE_ flux equation alone predicted that slow mCa^2+^ influx could have occurred via CHE_m_ this is unsustainable if [H^+^]_m_ < [H^+^]_e_. It is likely that voltage-dependent transport of net Ca^2+^ inward is mostly responsible if there is at least a partially maintained ΔΨ_m_ (Nernst potentials) despite mCa^2+^ extrusion via CHE_m_. Interestingly, under the condition of a fully polarized ΔΨ_m_ (no DNP and no OMN) (Figure 4A–D), [Ca^2+^]_m_ did not rise as it did in the presence of DNP (Figure 5A,B) when pH_m_ was maintained (Figure 7A,B). This suggests that the secondary, slow uptake of mCa^2+^ is indirectly related to H^+^ pumping due to the decline in [H^+^]_m_ to support the *pmf*; the additional, slow mCa^2+^ uptake by the MCU occurs because of the remaining charge gradient (ΔΨ_m_) and Ca^2+^ chemical gradient.

In contrast, in the presence of OMN the kinetics of the delayed, slow mCa^2+^ efflux via CHE_m_ under conditions of reduced ΔΨ_m_ and low pH_m_ are different. Our estimates of ΔΨ (Huang et al., 2007) of -60 to -70 mV at 700 s with OMN present are much lower than without OMN; this is likely due to dissipation of both ΔpH_m_ and ΔΨ_m_ gradients because H^+^ pumping by complex V to support ΔpH_m_ (and ΔΨ_m_) was blocked. With OMN present, we estimated Nernst potentials of +13 and +6 mV, respectively, for Ca^2+^ and H^+^ (calculated at 700 s). Based on these Nernst potentials the driving forces for both Ca^2+^ and H^+^ would remain inward with OMN present, although their Nernst potentials are reversed compared to those in the absence of OMN. With the slow inward driving force for H^+^, unmatched by H^+^ pumping at complex V, pH_m_ approached pH_e_ and net [Ca^2+^]_m_ became lowered due to CHE_m_. Because inhibiting the MCU with Ru360 caused a robust increase in [Ca^2+^]_e_, this indicated the Ca^2+^ effluxed via CHE_m_ re-enters via the MCU unless this pathway is blocked. Under the unique condition of collapsed ΔΨ_m_ (100 μM DNP) and ΔpH_m_ gradients, the secondary, slow uptake of mCa^2+^ is absent (Figure 5A,B, black lines) so that the decline in [Ca^2+^]_m_ via CHE_m_ is fully observed (Figure 6A,B). Thus, a fall in pH_e_ strongly supports net mCa^2+^ efflux via CHE_m_ even though the Nernst potentials indicate continued slow mCa^2+^ influx (via MCU), which indeed occurs if there is remaining ΔΨ_m_. This means that net Ca^2+^ efflux due to CHE_m_ (Figure 1, 2 and Supplementary Figure S.6) can be exposed by blocking the MCU after the initial bolus of CaCl_2_ to prevent further mCa^2+^ uptake. CHE_m_ is predicted by the *J*_CHE_ equation to favor mCa^2+^ efflux in exchange for mH^+^ influx based on matrix and buffer ion concentrations obtained with OMN present (Supplementary Figure S.10). Our prediction assumes that Ca^2+^ is exchanged for 2H^+^ with equal affinities for both cations, or a higher affinity for H^+^.

### Does Transient, Low Conductance mPTP Also Shuttle Ca^2+^ Across the IMM in These Experiments?

Inducing a partial ΔΨ_m_ depolarization was reported to cause a slow influx of mCa^2+^ through low conductance mPTP opening (Saotome et al., 2005). CsA prevented both an increase in mCa^2+^ and the release of the small molecule calcein during simulated ischemia in cardiomyocytes suggesting that transient mPTP opening during ischemia allowed mCa^2+^ influx (Seidlmayer et al., 2015). In the present study adding CaCl_2_ in the presence of DNP or an acidic buffer caused falls in ΔΨ_m_, so could low conductance mPTP opening have contributed to the secondary, slow increase or decrease in _m_[Ca^2+^] we observed in the absence or presence of OMN? We doubt this for the following reasons: (1) ROS, adenine nucleotide levels, and other factors are believed to contribute to mPTP formation during IR injury. But in our study we did not utilize IR to induce increases in Ca^2+^ and ROS or decreases in pH_m_ or ΔΨ_m_; (2) Altering just the driving force for protons across the IMM using DNP or external pH to exchange Ca^2+^ ion for H^+^ ions is not compatible for a mechanism to cause or prevent formation of mPTP but it is for inducing mCHE activity; (3) Transient mPTP formation is controversial and based largely on the utility of calcein or other small particles to mark mitochondrial release of small molecules with free flowing ions such as Ca^2+^ (Petronilli et al., 1999); (4) CsA-sensitive transient mPTP opening in individual mitochondria of cardiac myocytes is quite rare even with elevated m[Ca^2+^] or exposure to H_2_O_2_ (Lu et al., 2016); (4) CsA, or its inhibition of the peptidyl prolyl *cis–trans* isomerase activity of cyclophilin D, has known and unknown effects on mitochondrial function that may be unrelated to mPTP formation (Giorgio et al., 2010). Some interpretations on effects of cyclophilin D, via CsA, may pertain to changes in Ca^2+^ flux due to mCHE rather than transitional mPTP opening.

### CSA Ceases Activation of CHE_m_

CsA unexpectedly stopped the secondary CaCl_2_-induced effects attributed to CHE_m_. CsA ceased all apparent CHE_m_ activity after adding CaCl_2_ when pH_e_ was 6.9 or 7.15, as assessed by measurements of extra-matrix [Ca^2+^]_e_, pH_m_, and ΔΨ_m_ (Supplementary Figures S.1A–C). CsA did not blunt the partial ΔΨ_m_ depolarization induced by DNP alone at pH_e_ 7.15, but did delay full ΔΨ_m_ depolarization induced by adding CaCl_2_ after DNP (Supplementary Figures S.7A,B). We do not believe the slow, attenuated decreases in extrusion of Ca^2+^ or slow fall in matrix pH observed in the presence of CsA are directly related to inhibition of permanent or transient mPTP opening. CsA did not directly prevent the ΔΨ_m_ depolarization that occurs during CHE_m_ or with addition of DNP alone. In the absence of CsA (Figure 1A–C), the observed changes in pH_m_, external [Ca^2+^]_e_, and ΔΨ_m_, induced by adding CaCl_2_ at extra-matrix pH 6.9, occurred very slowly over 25–30 min; this is indicative of slow cation exchange activity, not mPTP. Moreover, full ΔΨ_m_ depolarization was incomplete. CsA or its inhibition of cyclophilin D may obviate the conditions for matrix H^+^ influx or mCa^2+^ efflux as well as Ca^2+^ recycling via the MCU. CsA may prevent dissipation of the ΔpH gradient when the external pH is low. Since the results obtained in the presence of CsA are not compatible with preventing or delaying mPTP opening, the effects of CsA in this setting are unclear. Additional experiments will be needed to delineate the mechanism of CsA on preventing CHE_m_.

### Other Potential Limitations of the Study

One important limitation of our study is the lack of a selective inhibitor of CHE_m_ to aid in defining a more precise mechanism of action. Since the gene code for LETM1 and its protein sequence are known, point mutations (Tsai et al., 2014) and knockdowns (Jiang et al., 2013; Doonan et al., 2014) in mammalian models will be helpful to assess mechanisms and kinetics of this cation antiporter; but it remains unclear if LETM1 mediates CHE_m_ exclusively, or at all. Another limitation is that mitochondria were examined outside their normal milieu so that the contributions of ATP synthesis by glycolysis and ATP hydrolysis for cellular metabolic support could not be assessed. Experiments were conducted at room temperature at which metabolism would be lower and buffering capacity different than at 37°C. The activity of CHE_m_ during cardiac IR is unknown and mCa^2+^ efflux in cardiac mitochondria may occur primarily via the NCE_m_ and not CHE_m_. Nevertheless, induction of CHE_m_ could occur *in vivo* during IR injury under very specific circumstances of trans-IMM cationic imbalance. Evaluation of CHE_m_ activity in cardiac myocytes after IR injury should be helpful to design protective strategies using this mechanism.

## Conclusion

This study furnishes new insights into the bioenergetic and dynamic mechanisms in cardiac cell mitochondria of delayed, slow mCa^2+^ influx via the MCU, and mCa^2+^ efflux via the pH_m_-dependent CHE_m_. We demonstrate the kinetics of slow changes in mCa^2+^ loading/unloading that are linked to unblocked *vs*. blocked ATP_m_ hydrolysis to decrease *vs*. increase pH_m_, respectively, after partial depolarization by DNP. We found that after an initial CaCl_2_ bolus there is slow mCa^2+^ influx (Ca^2+^ leak) through a Ru360-sensitive pathway if H^+^ pumping counteracts a H^+^ leak; however, there is net slow mCa^2+^ efflux that overrides ΔΨ_m_-mediated Ca^2+^ influx that is activated via CHE_m_ if there is a high ΔpH_m_ gradient. In cardiac mitochondria, the rapid and slow mode of uptake of mCa^2+^ appears to be dependent primarily on the trans-membrane [Ca^2+^] and ΔΨ_m_ gradients if outward H^+^ pumping counteracts inward H^+^ entry. In contrast, slow extrusion of mCa^2+^ by CHE_m_ appears to be dependent primarily on the [ΔH^+^]_m_ gradient induced by H^+^ influx/leak by DNP or by an acidic pH_e_. Importantly, if NCE_m_ and NHE_m_ are inactivated, blocking complex V might prevent delayed Ca^2+^ overload and instead stimulate Ca^2+^ extrusion via CHE_m_ if there is an inward H^+^ leak. In intact cells, this can also serve to preserve TCA cycle-generated ATP, i.e., substrate level phosphorylation. Such passive homeostatic balance of Δ[Ca^2+^]_m_ may occur during cardiac injury when there is mCa^2+^ loading accompanied by declines in NADH redox state, pH_m_ and Ψ_m_. We conclude that the differences in the rate and magnitude of mCa^2+^ influx/efflux in partially depolarized mitochondria, in the presence or absence of F_O_F_1-_ATPase activity, can be ascribed to the underlying changes in *pmf* components, ΔpH_m_ and ΔΨ_m_, after rapid mCa^2+^ loading.

## Author Contributions

DS proposed the study and its initial design. JH conducted most experiments, carried out initial statistical analysis, constructed initial figures, and participated in design, interpretation and writing. AG, AB, CB, CN, and MB conducted supporting experiments. AC, W-MK, and RD participated in theoretical interpretation of the results and text editing. DS and AC supervised the team in subsequent experimental designs, interpretation of results, and manuscript construction and writing.

## Conflict of Interest Statement

The authors declare that the research was conducted in the absence of any commercial or financial relationships that could be construed as a potential conflict of interest.
