# Supplementary material for: Slow Ca2+ Efflux by Ca2+/H+ Exchange in Cardiac Mitochondria Is Modulated by Ca2+ Re-uptake via MCU, Extra-Mitochondrial pH, and H+ Pumping by FOF1-ATPase
Source: Front Physiol. 2019 Feb 4;9:1914. doi: 10.3389/fphys.2018.01914 (PMC6378946; doi:10.3389/fphys.2018.01914)
Supplement: Supplementary file 1 [file Data_Sheet_1.pdf]

SUPPLEMENTAL MATERIALS

Slow Ca^{2+} efflux by Ca^{2+}/H^+ exchange in cardiac mitochondria is modulated by Ca^{2+} re-uptake via MCU, extra-mitochondrial pH, and H^+ pumping by F_OF_1-ATPase

Johan Haumann,[1,8] Amadou K.S. Camara,[1,2,4,5] Ashish K. Gadicherla,[1,10] Christopher D. Navarro,[1] Age D. Boelens,[1,9] Christoph A. Blomeyer,[1,11] Michael R. Boswell,[1] Ranjan K. Dash,[6] Wai-Meng Kwok,[1,3,4,5] and David F. Stowe[1,2,4,6,7]

[1]Anesthesiology Research Division, Departments of [1]Anesthesiology, [2]Physiology, [3]Pharmacology and Toxicology, [4]Cardiovascular Center, [5]Cancer Center, Medical College of Wisconsin, Milwaukee, WI, USA, Milwaukee, USA, [6]Department of Biomedical Engineering, Medical College of Wisconsin and Marquette University, [7]Research Service, Veterans Affairs Medical Center, Milwaukee, WI, USA

Present addresses:
[8]Department of Anesthesiology and Pain Management, Maastricht University Medical Centre, University Pain Centre Maastricht, The Netherlands
[9]Department of Anesthesiology, Academic Medical Center, University of Amsterdam, The Netherlands
[10]Institute of Clinical Medicine, University of Oslo, Oslo, Norway
[11]Department of Anesthesia and Critical Care, University Hospital of Wuerzburg, Germany

S.1 Materials and methods

S.1.1. Mitochondrial isolation – Guinea pig heart mitochondria were isolated as described before [1-5]. Guinea pigs (250-350 g) were anesthetized by intraperitoneal injection of 30 mg ketamine; 700 units heparin was given for anticoagulation. Hearts (n >90) were excised and minced to approximately 1 mm^3 pieces in ice-cold isolation buffer containing in mM: mannitol 200, sucrose 50, KH_2PO_4 5, 3-(N-morpholino) propanesulfonic acid (MOPS) 5, EGTA 1, BSA 0.1%, pH 7.15 (adjusted with KOH). The minced heart was suspended in 2.65 ml buffer with 5U/ml protease, and homogenized at low speed for 20 s; next 17 ml isolation buffer was added, and the suspension was again homogenized for 20 s. The suspension was centrifuged at 8000 *g* for 10 min. The supernatant was discarded and the pellet was suspended in 25 ml of isolation buffer, and centrifuged at 900 *g* for 10 min. The supernatant was centrifuged once more at 8000 *g* to yield the final mitochondrial pellet, which was suspended in 0.5 ml isolation buffer and kept on ice. The mitochondrial protein concentration was measured using the Bradford method [6], and diluted to 12.5 mg mitochondrial protein/ml with isolation buffer. All chemicals were obtained from Sigma-Aldrich (St. Louis, MO) unless noted otherwise.

S.1.2. Fluorescence measurements – Fluorescence spectrophotometry was used to measure matrix $[Ca^{2+}]_m$, NADH, pH_m, and mitochondrial membrane potential ($\Delta\Psi_m$) (Qm-8, Photon Technology International, Birmingham, NJ) [1,3,4,7]. A subset of isolated mitochondria (5 mg/ml) was incubated for 20 min at room temperature (25°C) with 5 µM indo-1 acetyl methyl ester (AM) to measure $[Ca^{2+}]_m$ or 5 µM 2'7'-bis-(2-carboxyethyl)-5'(and 6-) carboxyfluroescein AM (BCECF) to measure pH_m (Invitrogen, Carlsbad, CA), followed by suspension in 25 ml isolation buffer and centrifugation at 8000 *g*. The AM form of the dye is taken up into the mitochondrial matrix where it is de-esterified, so that the dye is retained in the matrix. The dye-loaded pellet was resuspended in 0.5 ml isolation buffer, and the protein concentration was measured again and diluted to 12.5 mg mitochondrial protein/ml. NADH was measured using autofluorescence and $\Delta\Psi_m$ was measured using rhodamine 123 (R123). Mitochondria were kept on ice for the duration of the studies. All studies were conducted at room temperature.

S.1.3. Measurement of $\Delta\Psi_m$ – $\Delta\Psi_m$ was assessed in mitochondria from 10 hearts in 4-5 replicates per heart by adding 50 nM rodamine-123 (R123, Calbiochem, San Diego, CA) to the buffer [7]. At an excitation wavelength (λ_{ex}) of 503 nm the change in fluorescence was measured at the emission wavelength (λ_{em}) of 527 nm. R-123 uptake is dependent on $\Delta\Psi_m$ [7]. As the dye is taken up, the fluorescence signal decreases as the dye autoquenches; thus, a decrease in $\Delta\Psi_m$ is represented by an increase in signal. Mitochondria energized with PA were considered fully polarized (0%), whereas the signal

after adding CCCP represented complete depolarization (100%). Actual $\Delta\Psi_m$ is not directly proportion to R-123 fluorescence and so only estimates $\Delta\Psi_m$. See text for use of TMRM to assess $\Delta\Psi_m$.

S.1.4. Measurement of matrix ionized [Ca^{2+}] – [Ca^{2+}]$_m$ was measured in Indo-1AM -loaded mitochondria from 14 hearts in 3-4 replicates per heart. Indo-1 is a fluorescent dye that binds to Ca^{2+} with a K_d tested to be approximately 240 nM. The λ_{em} shifts from 456 nm to 390 nm on binding to Ca^{2+} when a λ_{ex} of 350 nm is applied. The ratio between the two λ_{em}'s corrects for differences in the amount of dye taken up into mitochondria. Since the λ_{ex} and λ_{em} used for Ca^{2+} are the same as for NADH, the two NADH background λ_{em} signals were subtracted from the two λ_{em} indo-1 signals before calculating the ratio (R). The ratios obtained when all indo-1 becomes bound to Ca^{2+} (R_{max}) and when the lowest amount of Ca^{2+} is bound to indo-1 (R_{min}) were measured in energized mitochondria using 500 nM cyclosporine A and 500 µM $CaCl_2$ for R_{max}, and A23187 (Ca^{2+}-ionophore) and 2.5 mM EGTA for R_{min} [3]. [Ca^{2+}]$_m$ was calculated using the calibration formula [8]:

$$[Ca^{2+}]_m \text{ (nM)} = K_d \bullet (R-R_{min})/(R_{max}-R) \bullet S_{456}$$

K_d is the binding constant, and S_{456} is the ratio of fluorescence intensities during unsaturated and saturated Ca^{2+} at the 456 nm λ_{em}. Their ratio was measured to be 1.35. The Ca^{2+} signals were normalized to the averaged [Ca^{2+}]$_m$ over all experiments at time point t = 10 s (see *Experimental Protocol*), which was calculated to be approximately 80 nM. A 0.15 decrease in pH increases the K_d negligibly by about 9 nM [9]. ADP and ATP do not differentially alter light transmission at the λ_{ex} and λ_{em} spectra for indo-1, Fura 4F or an alternative Ca^{2+} fluorescent probe Rhod-2 (data not shown). To validate mCa^{2+} measurements by indo-1 AM, rhod-2 AM was substituted in some experiments (data not shown). The K_d for indo-1 increases with decreases in pH from 7.4 to 5.5, whereas K_d is not altered in the range of pH between 7.4 and 8.0 [10,11]. Any increase in K_d would increase proportionally the measured [Ca^{2+}]$_m$. Thus in Fig. 6A,B the effective decrease in [Ca^{2+}]$_m$ with a decrease in pH from approximately 7.25 to 7.05 might actually underestimate the fall in [Ca^{2+}]$_m$ that occurred over time because a small increase in K_d would counter the fall in [Ca^{2+}]$_m$. [Ca^{2+}]$_e$ was assessed using non AM probes.

S.1.5. Measurement of mitochondrial redox state – Mitochondria from 8 hearts in 3-4 replicates per heart were used to measure NADH autofluorescence. Unlike NAD, NADH molecules have natural fluorescence properties that can be monitored [12]. Therefore, an increase in the signal reflects an increase in the ratio of NADH to NAD^+, i.e. a shift to a more reduced state. The emission spectrum of NADH is broad, and peaks at λ_{em} 456 nm and λ_{ex} 350 nm. To correct for differences in total NADH and NAD^+ pool sizes, the ratio of λ_{em} 456/390 nm was measured. In addition to providing data on the mitochondrial redox state, the raw NADH data was used to correct for the background autofluorescence measured by the indo-1 fluorescence probe for [Ca^{2+}]$_m$ [3,8].

S.1.6. Measurement of matrix pH – Matrix pH was measured in BCECF-AM-loaded mitochondria from 10 hearts in 3-4 replicates per heart at λ_{ex} 504 nm and λ_{em} 530 nm. BCECF is a fluorescent probe that becomes less fluorescent in an acidic environment; thus an increase in signal indicates matrix alkalinization and a decrease in signal indicates matrix acidification. The measured signals were normalized for each group to their average photon count at the steady state seen after adding $CaCl_2$ or vehicle to correct for differences in signal strength and dye uptake. The measured signal was converted to pH units by measuring the BCECF signal from tritonized (1% triton X-100) mitochondria incubated in BCECF in buffers with known pH (7.00, 7.15 and 7.25) [3]. This gave a linear relationship, which enabled calculation of pH_m from the signal intensity. Because the wavelengths used for BCECF measurements did not overlap with the NADH auto-fluorescence signals, the matrix NADH and pH_m measurements were conducted in the same mitochondrial preparation.

S.1.7. Ruthenium 360 to assess uptake and re-uptake of Ca^{2+}_m – Because [Ca^{2+}]$_m$ was observed to slowly increase after adding $CaCl_2$ (Fig. 5, main text), an inhibitor of mitochondrial Ca^{2+} uptake via the mitochondrial Ca^{2+} uniporter (MCU), Ru360 (1 µM), was given 60 s after 10 or 25 µM $CaCl_2$ in 4 hearts to determine any effect on subsequent Ca^{2+} uptake assessed by indo-1 AM (matrix Ca^{2+}) and free indo-1 (external Ca^{2+}) See *S.2.4* and **Fig. S.6** below; see also Figs. 1, 2 (main text).

S.1.8. Measurement of extra-mitochondrial ionized [Ca^{2+}]$_e$ by Indo-1 or Fura 4F – Buffer Ca^{2+} containing mitochondria was assessed with free indo-1 from 5 hearts and with Fura 4F from 10 hearts in 4-5 replicates per heart using fluorescence spectrophotometry to assess matrix Ca^{2+} flux in the presence

or absence of DNP and OMN or changes in matrix pH, respectively. Addition of ADP or ATP did not interfere with the excitation or emission spectral characteristics of either fluorescent probe (data not shown). K_d's for Fura-4 were corrected for pH: 0.88 µM (6.9); 0.68 µM (7.15); 0.43 µM (7.6).

S.1.9. Measurement of mitochondrial O_2 consumption – Mitochondrial O_2 consumption rate (respiration) was measured from 4 hearts with 2-3 replicates per heart using a Clark-type O_2 electrode (System S 200A; Strathkelvin Instruments, Glasgow, UK) as we have described before [1,3,13]. Functional integrity of mitochondria was determined by the respiratory control index (RCI), defined here as the ratio of state 3 (after added ADP) to state 4 respiration (before adding ADP). Only mitochondrial preparations with RCIs ≥15, measured with pyruvic acid (PA), were used to conduct further experiments. See *S.2.2*; data after adding DNP are shown in **Fig. S.2**.

S.1.10. Measurement of ATP concentration – Mitochondrial [ATP] was estimated from ATP consumption in the total mitochondrial buffer using an ATP bioluminescent assay kit (Sigma-Aldrich): ATP+luciferin → luciferyl adenylate + PP_i; luciferyl adenylate + O_2 → oxyluciferin + AMP + light (proportional to ATP consumption). To do so, mitochondria from 20 hearts were suspended in experimental buffer and the detailed protocol as described above (Fig. 3, main text) was followed, with the exceptions that mitochondria were added at t = -120 s, then PA at t = 0, next DNP (0, 10, 20, 30 or 100 µM), and then $CaCl_2$ (0, 10, or 25 µM) at the same time points. CCCP was not added in these experiments. At specific time points all proteins were precipitated by adding 100 µl of 70% perchloric acid (Sigma-Aldrich) to quench all reactions. The obtained aliquot was centrifuged for 1 min at 50 *g*, 750 µl of the supernatant was collected, and the acidity was reversed by adding 180 µl of 5 M KOH. ATP was measured in buffer containing 200 mM MOPS, 2 mM EGTA, 3 mM $MgCl_2$, 0.3 mM D-luciferin, and 1.25 mg/ml luciferase at pH 7.20 (adjusted with KOH). Samples of 2.4 µl were added to 97.5 µl buffer, the solution was mixed, and luminescence was measured using a luminometer (Turner Biosystems). Total buffer [ATP] was calculated from the calibration curve generated using 62.5 nM, 125 nM, and 1250 nM [ATP] standards. Mitochondrial [ATP] was estimated from the final mitochondrial protein concentration (8.8 µg/ml) and the ratio of mitochondrial water to protein [14] as follows:

$$\text{Final calculated mitochondrial [ATP]} = \frac{\text{([ATP]}(10^{-9}\text{ M})(10^{-3}\text{ L/ml})}{(8.8\ \mu\text{g/ml})(66.4/25.0\text{ nl}/\mu\text{g})(10^{-9}\text{ L/nl})}$$

where mitochondrial protein mass = 0.25 of the total mitochondrial mass; mitochondrial water mass = 0.664 of the total mitochondrial mass; 66.4 g mitochondria H_2O/g sample = 66.4 nl mitochondria H_2O/µg because 1 µg H_2O = 1 nl H_2O; for example: if buffer [ATP] is 50 nM, then calculated mitochondrial [ATP] = 2.15 mM based on the estimated overall dilution factor of $4.3 \cdot 10^4$. Results shown in Fig. 9 (main text). (See below for assessment of ATP/ADP ratios by HPLC and luminometry.)

S.1.11. Measurement of [ADP] and [ATP] using HPLC – Mitochondrial [ATP] and [ADP] in the experimental buffer were determined in 5 hearts using the method of Liu et al. [15]. Briefly, mitochondria were treated as described previously (in the measurement of ATP synthesis/hydrolysis section) and the aliquot after perchloric acid precipitation was used to measure [ATP] and [ADP]. 200 µl of the supernatant was dried under a steady N_2 stream and resuspended in 20 µl mobile phase A consisting of 60 mM K_2HPO_4 and 40 mL KH_2PO_4. The mixture was then injected into the HPLC column and elution monitored at 254 nm. Standard mixtures of 0.125, 0.25, 0.5 and 1.0 mM ADP or ATP were used for calibration and to calculate [ADP] and [ATP] using area under the curve for extrapolation. Data are expressed as a ratio ([ADP]/[ATP]). See *S.2.9* for results using this method.

S.1.12. Measurement of ADP/ATP ratio using luminometry – An ADP/ATP ratio assay bioluminescent kit (ab65313; Abcam®) was used to calculate ADP/ATP ratios to complement measures of [ADP] and [ATP] obtained using HPLC. Mitochondria from 4 hearts were prepared exactly as in the HPLC method, except that instead of perchloric acid to quench all enzyme activity, 100 µM oligomycin (OMN) was used to block F_oF_1ATPsynthase/hydrolysis. Briefly, nucleotide releasing buffer and ATP monitoring enzyme first were added to a 96 well microtitre plate and then 50 µl of the mitochondrial suspension. After 1 min ATP levels were recorded using a luminometer (Data A). To record ADP levels, the ATP levels were recorded again after 10 min (Data B), and again 1 min after adding ADP converting enzyme (Data C). The ATP/ADP ratio was calculated as (Data B-Data C)/Data A. See *S.2.9* for results using this method.

S.2. Supplemental results and comments

S.2.1. Effect of cyclosporine A (CsA) on CHE_m – CsA (500 nM) appeared to cease CHE directly, or indirectly through inhibition of cyclophilin D by CsA. In the presence of CsA at an external pH_e of 6.9, adding 40 μM $CaCl_2$ did not result in a slow fall in matrix pH_m or a slow increase in extra-matrix $[Ca^{2+}]_e$ (also at pH_e 7.15) this was accompanied by the lack of a slow fall in $\Delta\Psi_m$ (**Fig. S.1A-C**).

S.2.2. Mitochondrial respiration is accelerated by DNP – Without added $CaCl_2$ or OMN, DNP increased the state 2 respiratory rate (**Fig. S.2**) from 18 (0 DNP=DMSO) to 28 (10 μM DNP), 33 (20 μM DNP), 72 (30 μM DNP), and 80 nmol•mg^{-1}•min^{-1} (100 μM DNP). State 3 respiration was little affected by DNP alone while state 4 respiration was accelerated with increasing DNP to approximately the levels observed in state 2. Respiratory Control Indices (RCI = state 3/state 4 were: 18 (DMSO), 10 (10 DNP), 7 (20 μM DNP), 4 (30 μM DNP), and 3 (100 μM DNP), indicating significant uncoupling of oxidative phosphorylation by DNP. In the presence of 20 μM DNP and 10 μM $CaCl_2$, uncoupling was greater (RCI = 1.8) compared to no added $CaCl_2$ (RCI = 7); this was presumably due to the larger decline in $\Delta\Psi_m$ with added $CaCl_2$, which enhances respiration in an attempt to restore $\Delta\Psi_m$.

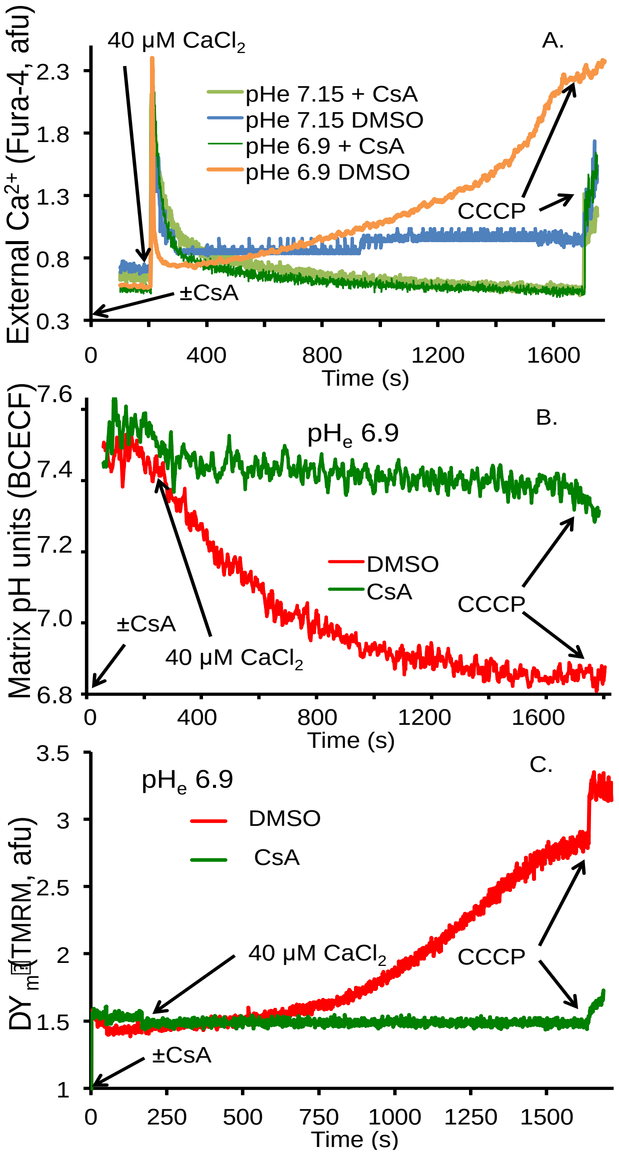

Fig. S.1. Effects of CsA on blocking apparent CHE_m-mediated H^+ influx (pH_m) (A), Ca^{2+} efflux ($[Ca^{2+}]_m$ (B), and $\Delta\Psi_m$ (C) after adding $CaCl_2$. Extra-mitochondrial pH_e was 6.9 or 7.15. Representative tracings from 3 experiments for each fluorescent probe. Compare with Fig. 1 (main text)

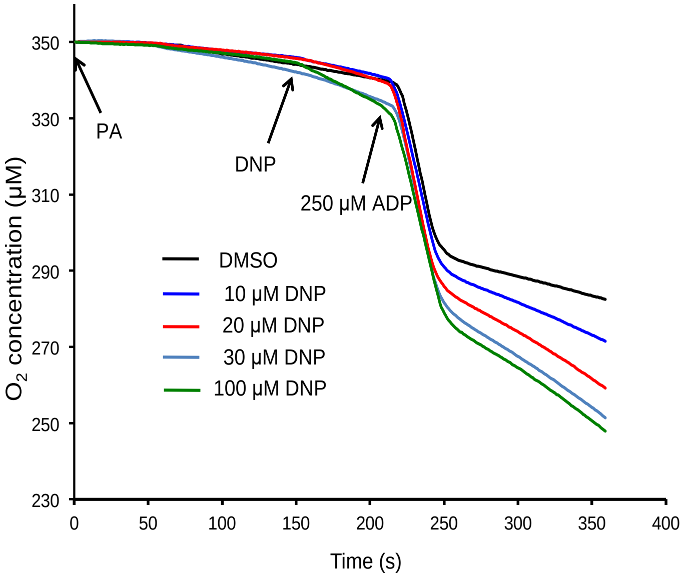

Fig. S.2. O_2 concentration in buffer containing mitochondria from 4 hearts energized with pyruvic acid (PA) and treated with either of four concentrations of DNP followed by ADP (beginning of state 3; exhaustion of ADP = beginning of state 4). Note that DNP accelerated the fall in O_2 consumption during states 2 and 4 and slowed it slightly during state 3. Note also that state 2 respiration increased even more when 10 μM $CaCl_2$ was given with 20 μM DNP, which nearly collapsed $\Delta\Psi_m$ (Fig. 4, main text).

*S.2.3. $\Delta\Psi_m$ (**Fig. S.3**), $[Ca^{2+}]_m$ (**Fig. S.4**), and pH_m (**Fig. S.5**) as functions of DNP before (215 s), early (275 s) and late (700 s) after adding $CaCl_2$.* Data are displayed below:

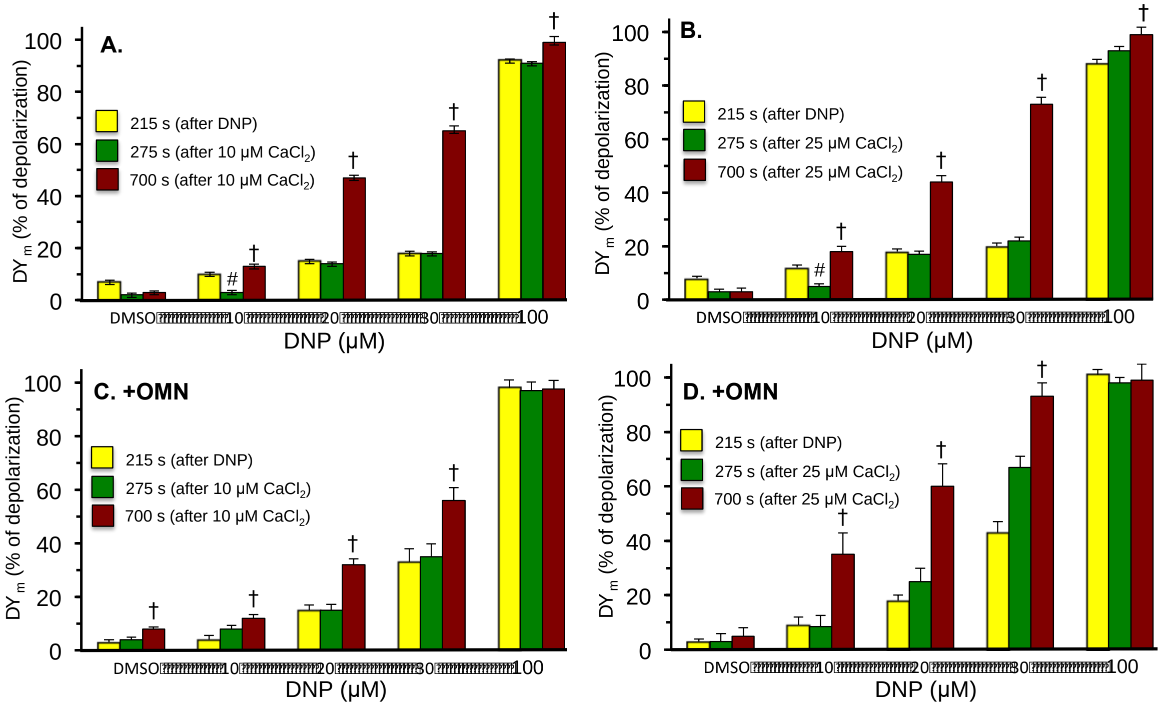

Fig. S.3. $\Delta\Psi_m$ as a function of [DNP] early (215 s), mid (275 s), and late (700 s) after adding either 10 or 25 μM $CaCl_2$ with or without oligomycin (OMN). Bar graph data summarizes timeline data furnished and described in Fig. 4. Data from 10 hearts. See Fig. 4 (main text) for changes in $\Delta\Psi_m$ over time and for statistical notation.

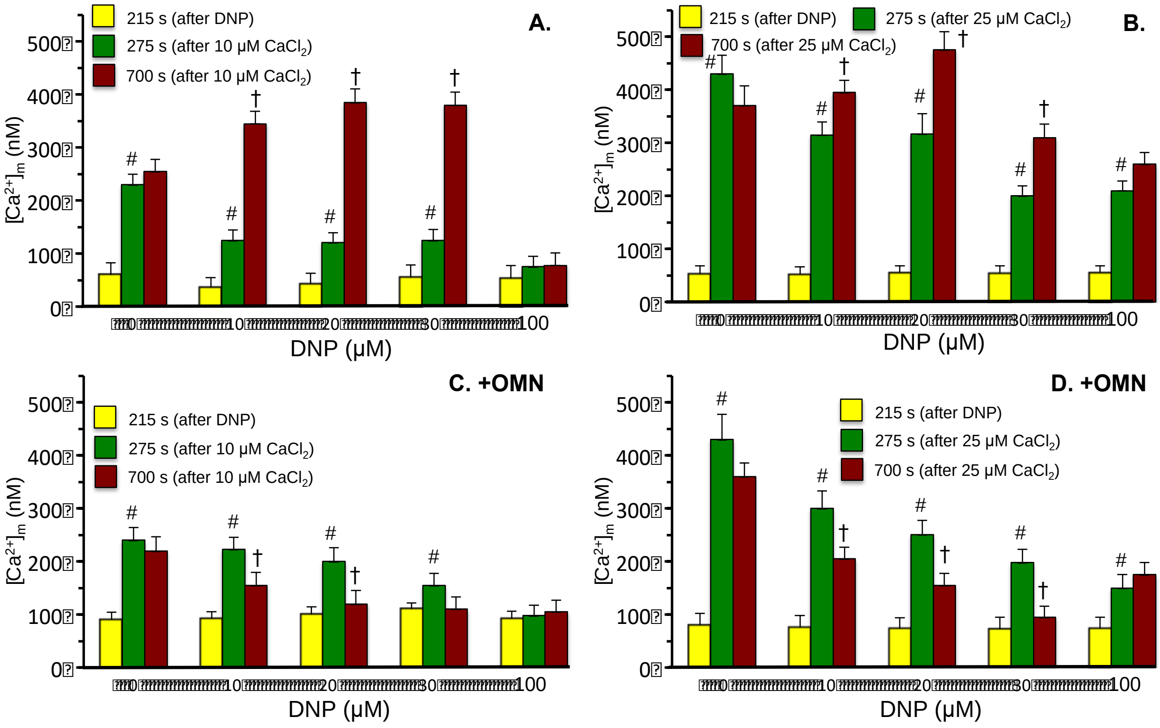

Fig. S.4. $[Ca^{2+}]_m$ as a function of DNP early (215 s), mid (275 s), and late (700 s) after adding either 10 or 25 μM $CaCl_2$ in the presence or absence of oligomycin (OMN). Bar graph data summarizes timeline data furnished and described in Figs. 5A,B and 6A,B). Data from 14 hearts. See Fig. 4 (main text) for statistical notation.

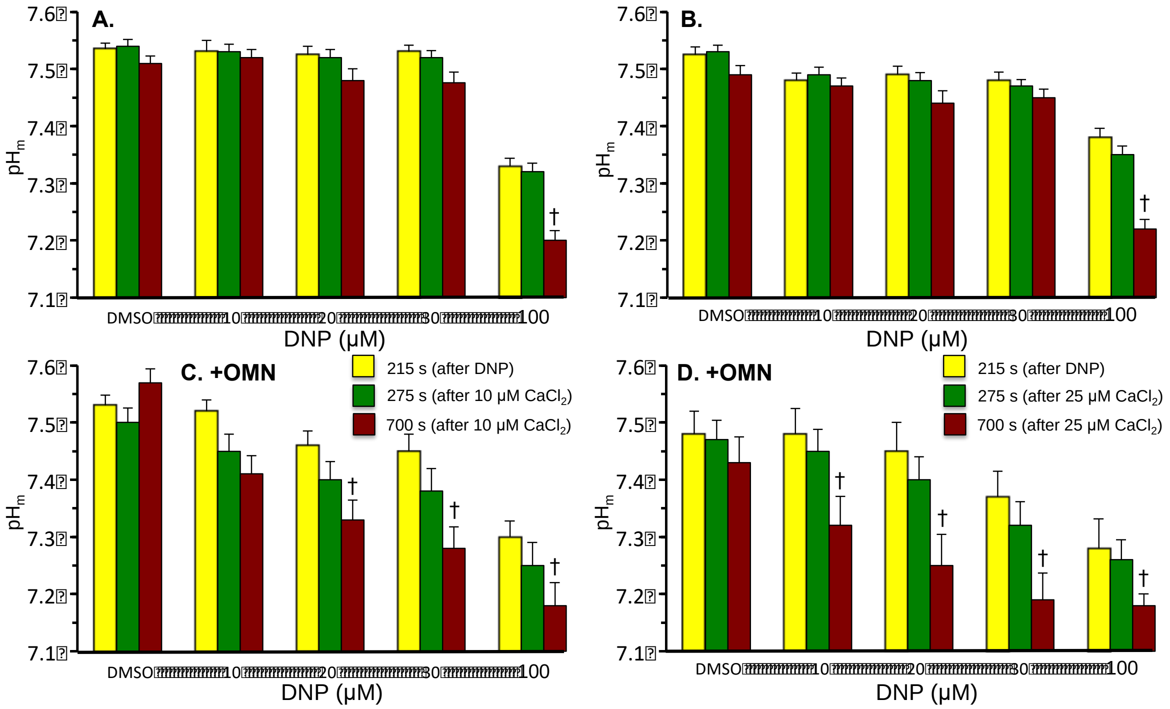

Fig. S.5. pH$_m$ as a function of DNP early (215 s), mid (275 s, and late (700 s) after adding either 10 or 25 μM $CaCl_2$ in the presence or absence of oligomycin (OMN). Bar graph data summarizes timeline data furnished and described in Fig. 7. Data from 10 hearts. See Fig. 4 (main text) for statistical notation.

S.2.4. Slow uptake of $Ca^{2+}$from external buffer is altered by blocking complex V and MCU – Adding 25 μM $CaCl_2$, and less so 10 μM $CaCl_2$, rapidly increased buffer $[Ca^{2+}]_e$ (**Fig. S.6**), which then slowly decreased as Ca^{2+} was taken up by mitochondria. Adding DNP slightly retarded the fall in $[Ca^{2+}]_e$ over time in the absence of OMN (**A**) but largely reduced the fall in $[Ca^{2+}]_e$ in the presence of OMN indicating less Ca^{2+} uptake (**B**). This indicated DNP slightly reduced net mCa^{2+} uptake with OMN and largely enhanced net mCa^{2+} uptake without OMN. This suggests the slow secondary fall in observed $[Ca^{2+}]_m$ with OMN was due to slow efflux of Ca^{2+} via CHE_m in exchange for mH^+ influx at a slightly (10-20%) depolarized $\Delta\Psi_m$ (Fig. 4, main text) to counter mCa^{2+} influx via MCU (Fig. 6, main text). In the presence of 100 nM Ru360 given after adding $CaCl_2$, the declines in $[Ca^{2+}]_e$ ±OMN were arrested and followed by a slow increase in $[Ca^{2+}]_e$ over time. This indicates that Ru360 blocks MCU-mediated mCa^{2+} re-entry (Figs. 1,2 main text) and exposes slow mCa^{2+} efflux via CHE_m, particularly if complex V is blocked (+OMN).

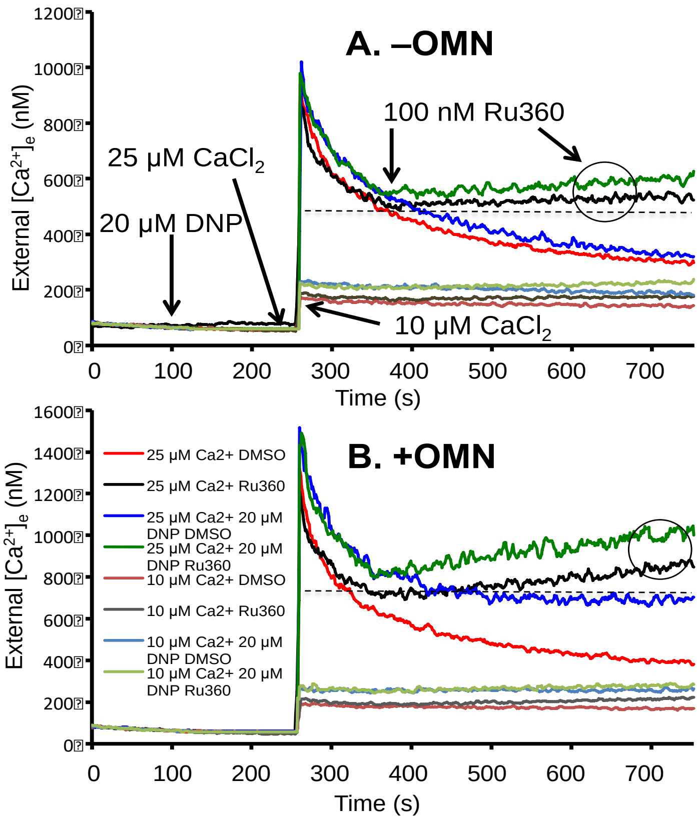

Fig. S.6. Changes in buffer (external, e) $[Ca^{2+}]_e$, assessed by indo-1 fluorescence in the presence of added DNP and $CaCl_2$ ± addition of 10 μM oligomycin (OMN) and/or 100 nM ruthenium 360 (Ru360). Representative data from 4 hearts.

Refer to Figs. 5,6 (main text) for changes in $[Ca^{2+}]_m$ over time. Buffer contained approxi-

mately 36-40 µM EGTA carried over from the isolation buffer.

S.2.5. Adding cyclosporine A (CsA) stops CHE$_m$ – CsA (500 nM) did not block a partial fall in $\Delta\Psi_m$ due to 30 µM DNP (**Fig. S.7**); in the absence of added $CaCl_2$, the fall in $\Delta\Psi_m$ was maintained for up to 25 min (**Fig. S.7A**). CsA delayed, but did not abolish, the $\Delta\Psi_m$ depolarization caused by DNP plus $CaCl_2$ (**Fig. S.7B**)

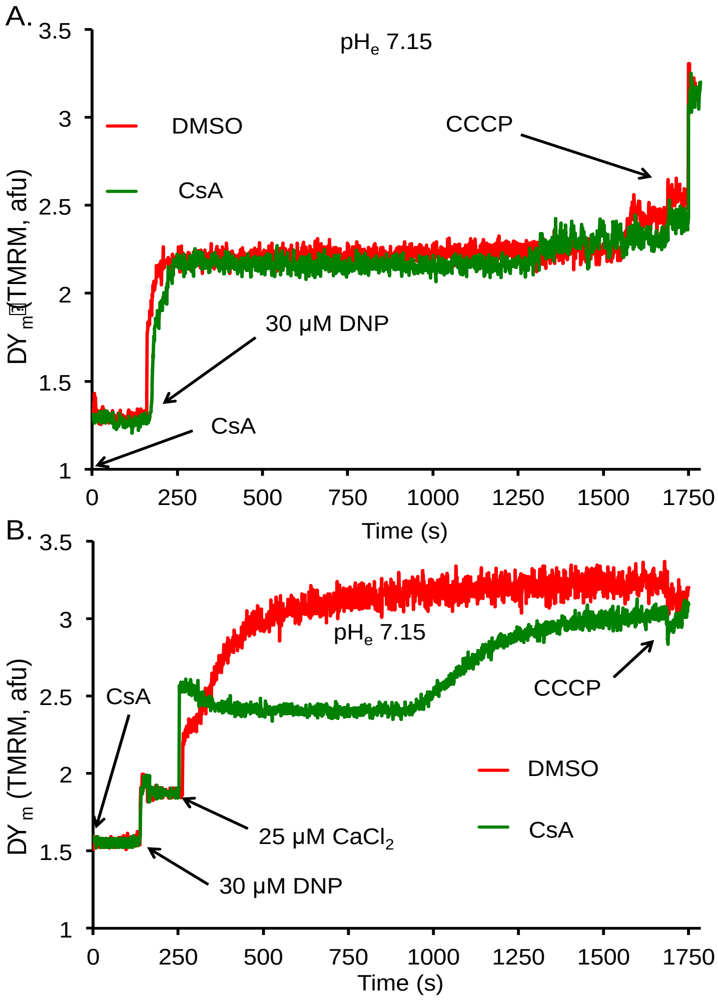

Fig. S.7. Lack of effect of CsA on steady-state DNP-induced partial $\Delta\Psi_m$ depolarization over time (A) and effect of CsA to delay $\Delta\Psi_m$ depolarization after adding $CaCl_2$ (B) in presence of DNP. Extra-matrix pH$_e$ was 7.15 in absence of OMN. Representative tracings from 4 experiments.

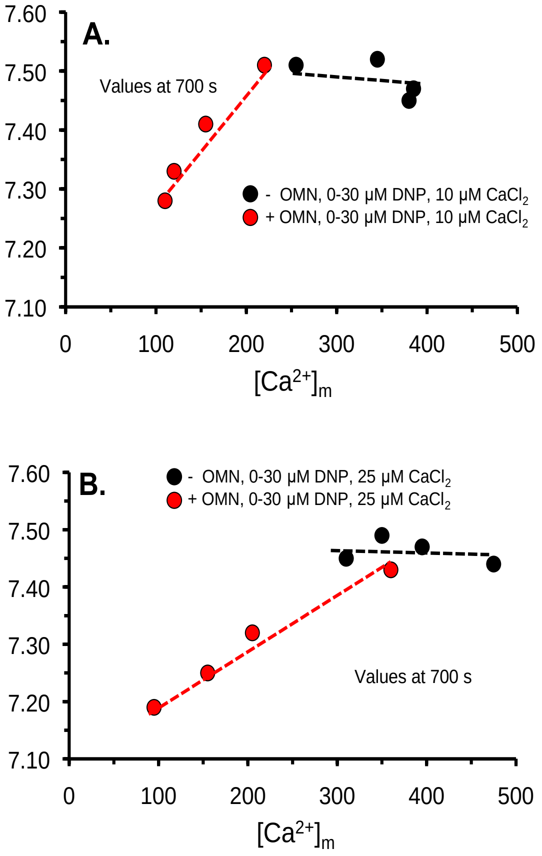

Fig. S.8. Plots of pH$_m$ as a function of $[Ca^{2+}]_m$ at 700 s in the presence of 0, 10, 20, and 30 µM DNP with either 10 (**A**) or 25 µM (**B**) $CaCl_2$ + OMN or DMSO (no OMN). Note the interdependence of pH$_m$ and $[Ca^{2+}]_m$ only in the presence of OMN.

S.2.6. Matrix $[Ca^{2+}]_m$ is lower when matrix $[H^+]_m$ is higher (lower pH$_m$) after blocking complex V – The snapshot of pH$_m$ as a function of $[Ca^{2+}]_m$ at the time point of 700 s (**Fig. S.8**) shows the relationship between matrix Ca^{2+} and matrix pH at increasing concentrations of DNP in the presence, but not the absence, of OMN. In the absence of OMN, proton pumping by complex V counteracts the influx of protons due to the protonophore DNP.

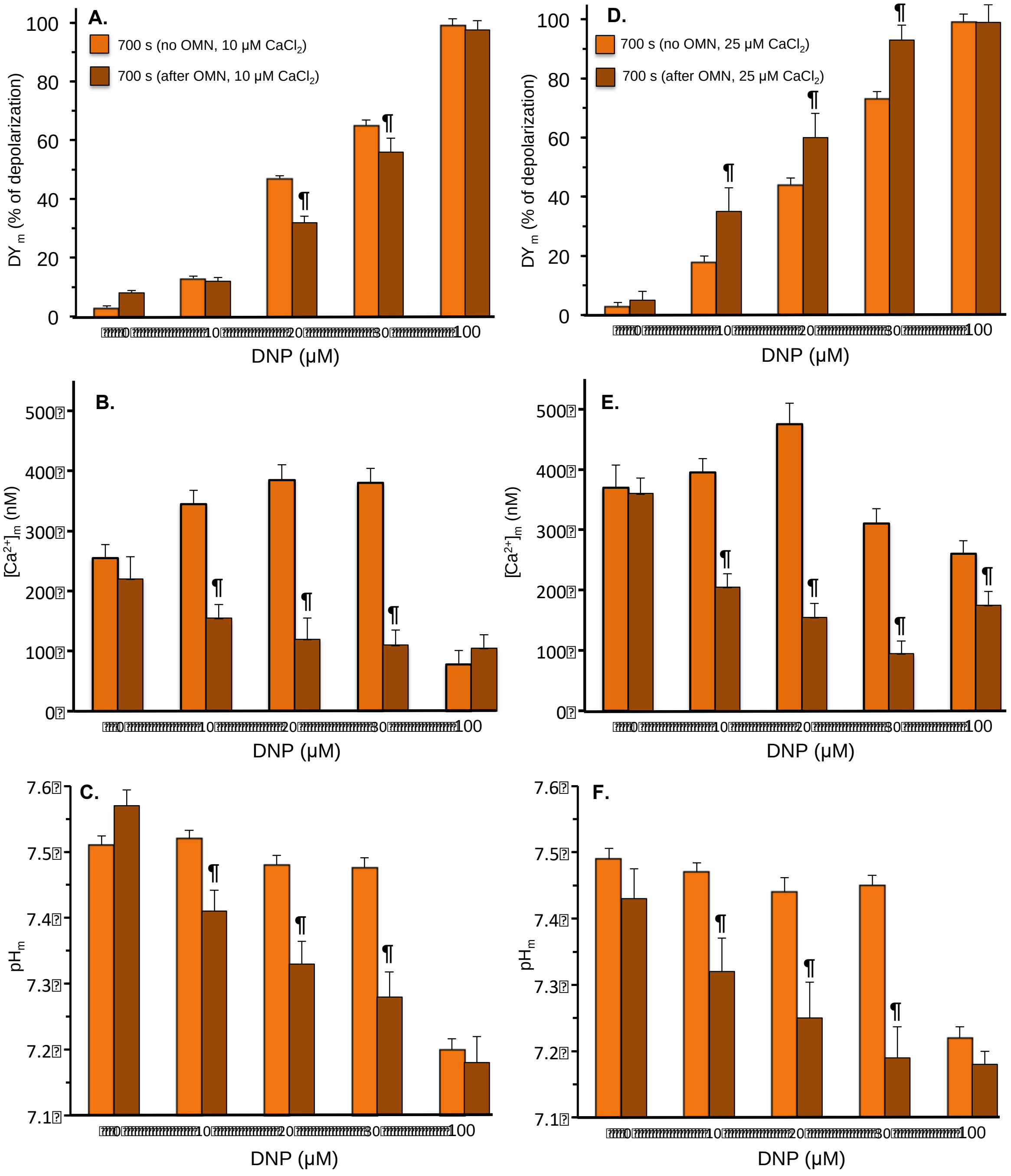

Fig. S.9. Comparison of changes in $\Delta\Psi_m$ (% of maximal depolarization, A,D) $[Ca^{2+}]_m$ (B,E) and pH_m (C,F) at t = 700 s in the presence or absence of OMN to block complex V. Data are rearranged from the 700 s (brown bars) of **Figs. S.3-5**. For *P*<0.05: ¶ plus OMN *vs*. no OMN (DMSO).

S.2.7. Contrasting time-dependent values for $\Delta\Psi_m$, $[Ca^{2+}]_m$, & pH_m dependent on block of complex V

Blocking H^+ pumping from complex V with OMN elicited pronounced effects on $\Delta\Psi_m$, $[Ca^{2+}]_m$, and pH_m (**Fig. S.9**) compared to when H^+ pumping was allowed at complex V (dark *vs*. light brown bars). $[Ca^{2+}]_m$ was markedly lower when $\Delta\Psi_m$ was largely depolarized and when OMN was present. This is in marked contrast to the situation in which complex V was not blocked from pumping H^+, as shown by the maintenance of pH_m (except at 100 μM DNP) and much higher $[Ca^{2+}]_m$ despite a falling $\Delta\Psi_m$ in both 10 (A,B,C) and 25 μM (D,E,F) $CaCl_2$ groups.

S.2.8. Ca^{2+}-H^+ exchange: calculated Ca^{2+} flux rates – For the equilibration reaction: $Ca^{2+}_m + 2H^+_e \leftrightarrows Ca^{2+}_e + 2H^+_m$, we used the J_{CHE} rate expression of Tewari et al. [16]:

$$J_{CHE} = X_{CHE}\left(\frac{[H^+]_e^2[Ca^{2+}]_m - [H^+]_m^2[Ca^{2+}]_e}{\left(K_{Ca,CHE}\left([H^+]_e^2 + [H^+]_m^2\right) + [H^+]_e^2[Ca^{2+}]_m + [H^+]_m^2[Ca^{2+}]_e\right)}\right)$$

The exchange of Ca^+ for H^+ via the CHE was assumed to be electroneutral (n_{CHE} = 2), and hence the flux was considered independent of $\Delta\Psi_m$. Note that the direction of Ca^{2+} flux is estimated by the placement of the two terms in the numerator of the flux equation. Conditions were after additions of 20 µM DNP and 25 µM $CaCl_2$ ±OMN. Matrix (m) $[Ca^{2+}]_m$ and pH_m values were taken from the means obtained in the control (no OMN) and the OMN groups of Fig. S.4 and S.5 (pH converted to $[H^+]$) at the 275, 500 and 700 s time periods. Extra matrix (e) $[H^+]_e$ was 89 nM (pH_e 7.15) and $[Ca^{2+}]_e$ values were estimated from Fig. S.6A,B. The value of the Ca^{2+} binding constant ($K_{Ca,CHE}$) parameter was 4,800•10^{-9} M; the value for CHE activity (X_{CHE}) was 4.7 nmol•mg^{-1}•min^{-1} [16]. Based on ion gradients alone (i.e. no facilitated ion transport), **Fig. S.10** estimates there would be an efflux of Ca^{2+} mediated solely by CHE over time in the presence of OMN (DMSO) *vs.* influx of Ca^{2+} in the absence of OMN.

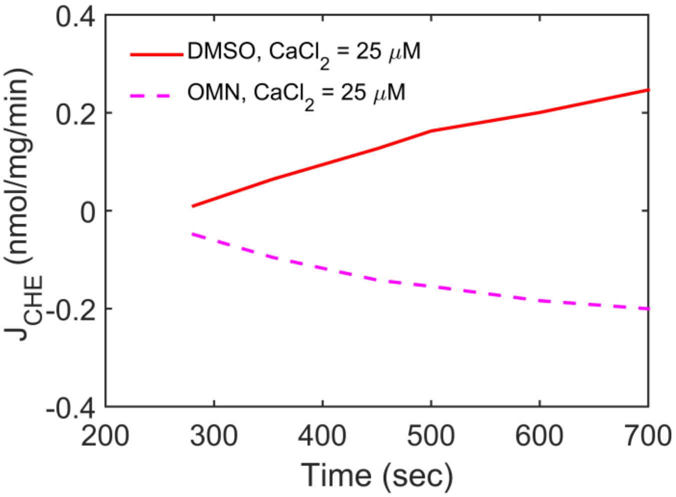

Fig. S.10. Calculated flux rate and direction of CHE_m-mediated Ca^{2+} entry into and out of the matrix based solely on the values obtained from Figs. S.3, S.5 in the presence or absence of OMN to block or permit H^+ pumping by complex V. Actual net Ca^{2+} flux is determined also by $\Delta\Psi_m$-dependent Ca^{2+} uptake by the MCU, and net H^+ flux by H^+ pumping. Plot depicts mCa^{2+} influx and efflux in the absence (DMSO) and presence of OMN, respectively.

S.2.9. Graded depolarization of $\Delta\Psi_m$ reduces the ATP/ADP ratio – We determined the ratio of ATP/ADP using either of two methods, HPLC *or* luminometry, respectively in energized, state 4 conditions. Mitochondria (in the absence of oligomycin) were treated with 0 or 20 mM DNP and 0, 10 or 25 µM $CaCl_2$, which stepwise reduced $\Delta\Psi_m$ (Fig. 4A,B, main text). We observed (data not displayed) that the ratio of ATP/ADP in the mitochondrial homogenate decreased proportionally from 2.9±0.4 *or* 7.4±1.1 (O DNP, 0 µM $CaCl_2$) to 1.8±0.2 *or* 3.9±2.1 (20 DNP, 0 $CaCl_2$), 1.1±0.2 *or* 2.9±1.9 (20 DNP, 10 µM $CaCl_2$), and 0.9±0.1 *or* 2.0±0.5 (20 DNP, 25 µM $CaCl_2$), using HPLC *or* luminometry, respectively. Thus these decreases in ATP relative to ADP accompanied the declines in $\Delta\Psi_m$ (Fig. 2A,B, main text).

S.2.10. Sources of ATP for hydrolysis – In the absence of exogenous nucleotides, the source of ATP for hydrolysis that occurred for up to 400 s in the absence of OMN is worth noting. The rapid O_2 consumption rate of 80 nmol•mg^{-1}•min^{-1}, measured after adding 100 µM DNP during state 2 respiration, (**Fig. S.2**) calculated to an oxidation rate of 27 nmol•mg^{-1}•min^{-1} for pyruvate; on a mole-to-mole basis, that would also be the rate of ATP produced (via GTP) by substrate level phosphorylation during conversion of succinyl-CoA to succinate in the TCA cycle. The more rapid O_2 consumption rate of 260 nmol•mg^{-1}•min^{-1} that was observed in the presence of $CaCl_2$ with DNP would produce ATP at a rate of 87 nmol•mg^{-1}•min^{-1}. We estimate that maximal ATP hydrolysis was about 3.3 mmol/L over 400 s (6.6 min), or about 500 nmol•mg^{-1}•min^{-1}. From these data we conclude that adequate matrix ATP stores, coupled with substrate level phosphorylation before (DNP alone) and during $\Delta\Psi_m < E_{REV\text{-}ATPase}$ (with added $CaCl_2$), were likely sufficient to supply adequate ATP for hydrolysis at complex V over at least a 500 s period

Table. Mitochondrial variables at DNP IC_{50} 700 s after initiating experiments with pyruvic acid.

	– OMN		+ OMN	
$CaCl_2$ (µM)	10	25	10	25
$\Delta\Psi_m$ (% maximal depolarization) (nM)	31	30	10	25
$d[Ca^{2+}]/dt$ (nM/s)	5	62	19	25
pH_m (units)	7.51	7.48	7.37	7.28

Values obtained by linear regression analysis at 15 µM DNP (range 0-30 µM DNP). All regression slopes were significantly greater (P<0.05) than zero.

References

[1] Heinen A, Camara AK, Aldakkak M, Rhodes SS, Riess ML, Stowe DF. Mitochondrial Ca^{2+}-induced K^+ influx increases respiration and enhances ROS production while maintaining membrane potential. Am J Physiol Cell Physiol. 292 (2007) C148-56.

[2] Riess ML, Kevin LG, McCormick J, Jiang MT, Rhodes SS, Stowe DF. Anesthetic preconditioning: the role of free radicals in sevoflurane-induced attenuation of mitochondrial electron transport in Guinea pig isolated hearts. Anesth Analg. 100 (2005) 46-53.

[3] Haumann J, Dash RK, Stowe DF, Boelens A, Beard DA, Camara AKS. Mitochondrial free $[Ca^{2+}]$ increases during ATP/ADP antiport and ADP phosphorylation: exploration of mechanisms Biophys J. 99 (2010) 997-1006.

[4] Aldakkak M, Stowe DF, Cheng Q, Kwok WM, Camara AK. Mitochondrial matrix K^+ flux independent of large-conductance Ca^{2+}-activated K^+ channel opening. Am J Physiol Cell Physiol. 298 (2010) C530-41.

[5] Riess ML, Camara AK, Heinen A, Eells JT, Henry MM, Stowe DF. K_{ATP} channel openers have opposite effects on mitochondrial respiration under different energetic conditions. J Cardiovasc Pharmacol. 51 (2008) 483-91.

[6] Bradford MM. A rapid and sensitive method for the quantitation of microgram quantities of protein utilizing the principle of protein-dye binding. Anal Biochem. 72 (1976) 248-54.

[7] Huang M, Camara AK, Stowe DF, Qi F, Beard DA. Mitochondrial inner membrane electrophysiology assessed by rhodamine-123 transport and fluorescence. Ann Biomed Eng. 35 (2007) 1276-85.

[8] Grynkiewicz G, Poenie M, Tsien RY. A new generation of Ca^{2+} indicators with greatly improved fluorescence properties. J Biol Chem. 260 (1985) 3440-50.

[9] Westerblad H, Allen DG. The influence of intracellular pH on contraction, relaxation and $[Ca^{2+}]_i$ in intact single fibres from mouse muscle. J Physiol. 466 (1993) 611-28.

[10] Lattanzio FA, Jr. The effects of pH and temperature on fluorescent calcium indicators as determined with Chelex-100 and EDTA buffer systems. Biochem Biophys Res Commun. 171 (1990) 102-8.

[11] Aldakkak M, Stowe DF, Heisner JS, Spence M, Camara AK. Enhanced Na^+/H^+ exchange during ischemia and reperfusion impairs mitochondrial bioenergetics and myocardial function. J Cardiovasc Pharmacol. 52 (2008) 236-44.

[12] Chance B, Cohen P, Jobsis F, Schoener B. Intracellular oxidation-reduction states in vivo. Science. 137 (1962) 499-508.
[13] Agarwal B, Dash RK, Stowe DF, Bosnjak ZJ, Camara AK. Isoflurane modulates cardiac mitochondrial bioenergetics by selectively attenuating respiratory complexes. Biochim Biophys Acta. 1837 (2014) 354-65.
[14] Vinnakota KC, Bassingthwaighte JB. Myocardial density and composition: a basis for calculating intracellular metabolite concentrations. Am J Physiol Heart Circ Physiol. 286 (2004) H1742-9.
[15] Liu H, Jiang Y, Luo Y, Jiang W. A simple and rapid determination of ATP, ADP and AMP concentrations in pericarp tissue of litchi fruit by high performance liquid chromatography. Food Technol Biotechnol. 44 (2006) 531-4.
[16] Tewari SG, Camara AK, Stowe DF, Dash RK. Computational analysis of Ca^{2+} dynamics in isolated cardiac mitochondria predicts two distinct modes of Ca^{2+} uptake. J Physiol. 592 (2014) 1917-30.
